# Grassland type and seasonal effects have a bigger influence on plant functional and taxonomical diversity than prairie dog disturbances in semiarid grasslands

**DOI:** 10.1002/ece3.9040

**Published:** 2022-07-13

**Authors:** Maria Gabriela Rodriguez‐Barrera, Ingolf Kühn, Eduardo Estrada‐Castillón, Anna F. Cord

**Affiliations:** ^1^ Chair of Computational Landscape Ecology, Institute of Geography Technische Universität Dresden Dresden Germany; ^2^ Department of Computational Landscape Ecology Helmholtz Centre for Environmental Research – UFZ Leipzig Germany; ^3^ Department of Community Ecology Helmholtz Centre for Environmental Research – UFZ Halle Germany; ^4^ Department of Geobotany and Botanic Garden/Institute for Biology Martin Luther University Halle‐Wittenberg Halle Germany; ^5^ German Centre for Integrative Biodiversity Research (iDiv) Halle‐Jena‐Leipzig Leipzig Germany; ^6^ Facultad de Ciencias Forestales Universidad Autónoma de Nuevo León Linares Nuevo Léon Mexico

**Keywords:** animal–plant interactions, disturbance, drylands, functional diversity, grassland ecosystems, plant diversity, prairie dogs, seasonal effects

## Abstract

1. Prairie dogs (*Cynomys* sp.) are considered keystone species and ecosystem engineers for their grazing and burrowing activities (summarized here as disturbances). As climate changes and its variability increases, the mechanisms underlying organisms' interactions with their habitat will likely shift. Understanding the mediating role of prairie dog disturbance on vegetation structure, and its interaction with environmental conditions through time, will increase knowledge on the risks and vulnerability of grasslands.
2. Here, we compared how plant taxonomical diversity, functional diversity metrics, and community‐weighted trait means (CWM) respond to prairie dog *C. mexicanus* disturbance across grassland types and seasons (dry and wet) in a priority conservation semiarid grassland of Northeast Mexico.
3. Our findings suggest that functional metrics and CWM analyses responded to interactions between prairie dog disturbance, grassland type and season, whilst species diversity and cover measures were less sensitive to the role of prairie dog disturbance. We found weak evidence that prairie dog disturbance has a negative effect on vegetation structure, except for minimal effects on C4 and graminoid cover, but which depended mainly on season. Grassland type and season explained most of the effects on plant functional and taxonomic diversity as well as CWM traits. Furthermore, we found that leaf area as well as forb and annual cover increased during the wet season, independent of prairie dog disturbance.
4. Our results provide evidence that grassland type and season have a stronger effect than prairie dog disturbance on the vegetation of this short‐grass, water‐restricted grassland ecosystem. We argue that focusing solely on disturbance and grazing effects is misleading, and attention is needed on the relationships between vegetation and environmental conditions which will be critical to understand semiarid grassland dynamics under future climate change conditions in the region.

## Graphical abstract

Understanding the mediating role of prairie dog disturbance on vegetation structure, and its interaction with environmental conditions through time, will increase knowledge on the risks and vulnerability of grasslands. Here, we compared how plant taxonomic and functional diversity metrics, along with community‐weighted trait means (CWM), respond to prairie dog disturbance across grassland types and seasons in a priority conservation semiarid grassland of Northeast Mexico. Our results provide evidence that prairie dog disturbance is less important than grassland type and that environmental effects have a stronger role than grazing and animal disturbances on vegetation.

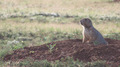

## 1 INTRODUCTION

Prairie dogs (*Cynomys* sp.) have evolved together with grasslands (Castellanos‐Morales et al., 2016; Goodwin, 1995; Seersholm et al., 2020) and provide key ecosystem engineering activities which make them valuable for grassland conservation (Davidson et al., 2010, 2012; Martinez‐Estevez et al., 2013). Their grazing and burrowing activities (from here on summarized as disturbances) directly and indirectly alter habitat structure crucial for the presence of other species such as the black‐footed ferret (*Mustela nigripes*; Kotliar et al., 2006) and the mountain plover (*Charadrius montanus*; Duchardt et al., 2019). They also prevent shrub encroachment (Ceballos et al., 2010; Ponce‐Guevara et al., 2016; Weltzin et al., 1997), maintain landscape heterogeneity (Bangert & Slobodchikoff, 2000; Davidson & Lightfoot, 2006; Gervin et al., 2019), increase fodder quality for cattle by reducing leaf age, which increases the plants nitrogen intake (Sierra‐Corona et al., 2015) and alter soil properties by increasing soil heterogeneity, infiltration rates and carbon storage (Barth et al., 2014; Martinez‐Estevez et al., 2013). Despite the positive impacts of prairie dogs on grasslands, their disturbance has shown to alter vegetation structure and characteristics considered priorities by ranchers, for example, by reducing biomass and cover of grasses as well as increasing cover of forb and annual species, resulting in reduced fodder quantity (Connell et al., 2019) and leading to the assumption that prairie dogs degrade grassland vegetation and compete with livestock. This in turn has led to prairie dogs being threatened by recreational shooting and poisoning (Miller et al., 2007). Although some conservation measures have been taken to preserve them (e.g., through agri‐environmental schemes and the designation of conservation areas), these have not been able to change the socio‐ecological views of local communities (Miller et al., 1994; SEMARNAT, 2018).

Many grasslands are disturbance‐adapted ecosystems (Gibson, 2009), on which small‐scale disturbances by herbivorous burrowing mammals (including prairie dogs) have played a fundamental role for vegetation structure (Davidson et al., 2012). As climate changes and its variability increases, the mechanisms underlying organisms' interactions with their habitat will likely shift (Baez‐Gonzalez et al., 2018). Understanding the mediating role of prairie dog disturbance on vegetation structure, and its interaction with environmental conditions through time, will increase knowledge on the risks and vulnerability of grasslands, allowing for future nature‐based solutions that can be applied to grassland management (Pörtner et al., 2021). Despite this, it is only recently that studies have started to include interactions between disturbance and multiple environmental conditions such as soil, precipitation, and temperature (Ahlborn et al., 2021; Buzhdygan et al., 2020; Jäschke et al., 2020), and very few have explored the role burrowing herbivorous mammal disturbances have on grasslands across such environmental conditions (Coggan et al., 2018).

Plant functional traits, that is, physiological, phenological, and morphological features, mediate between habitat disturbances and ecosystem functions and hence call for exploring trait variations within communities (Hanisch et al., 2020; Mouillot et al., 2013). Impacts on these traits would easily be ignored by looking solely into taxonomic diversity, which in most cases is not comparable between communities that are dissimilar or not complementary to each other, making generalization difficult (Chao et al., 2000), and loosing key information as to the direct effects of disturbance on biodiversity. Functional diversity indices summarize species' traits and their abundances via their distribution within the functional space, allowing to explore complementary characteristics between communities (Mouchet et al., 2010). Furthermore, the distribution of trait variations can be determined through environmental filtering and biotic filtering, for example, herbivory, which, acting as a filter, can increase or decrease the presence of certain traits (Mayfield & Levine, 2010; Zobel, 1997) and can thereby allow for the identification of niche processes (Mason et al., 2005; Mouillot et al., 2013; Villéger et al., 2008). Paired with community‐weighted means (CWM), we can analyze community trait diversity and their trait‐environment relationships (Funk et al., 2016; Miller et al., 2019).

Here, we focus on easy and quick field measured traits that have proven useful to identify vegetation responses to grazing, semiarid habitats and seasonality effects. Namely, leaf area, specific leaf area (SLA), and vegetative height are proxies for multiple ecosystem functions such as biomass production, fodder quality, soil fertility, water regulation, and competitive ability. Traits such as photosynthetic pathway, life history, and growth form relate to temperature, CO^2^ levels, available nutrients, water efficiency, as well as timing of maturity and survival strategies (Hanisch et al., 2020; Moles et al., 2009). For instance, plant responses to grazing have been shown to directly alter the distribution and variation of specific leaf area and height, favoring shorter species and lower SLA (Díaz et al., 2006; van der Plas et al., 2016). Short plants, with small SLA and leaf area, are associated with efficient water use (Blumenthal et al., 2020; Wellstein et al., 2017; Zhao et al., 2020). Furthermore, the selected traits are key to relate vegetation structure dynamics with ecosystem conditions within semiarid grasslands. These grasslands have evolved through droughts and disturbance regimes since the Pleistocene, developing high numbers of C4, perennial, and shrub species (Gibson, 2009). To the best of our knowledge, no other study has yet examined the functional relationship between different grassland types and the response of vegetation to disturbance by prairie dogs. Additionally, in this study, traits were measured directly in the field, allowing us to evaluate environmentally induced shifts on the selected traits (phenotypic plasticity; Nicotra et al., 2010). Furthermore, no study that we know of has analyzed how these relationships change over the seasons.

**FIGURE 1:**
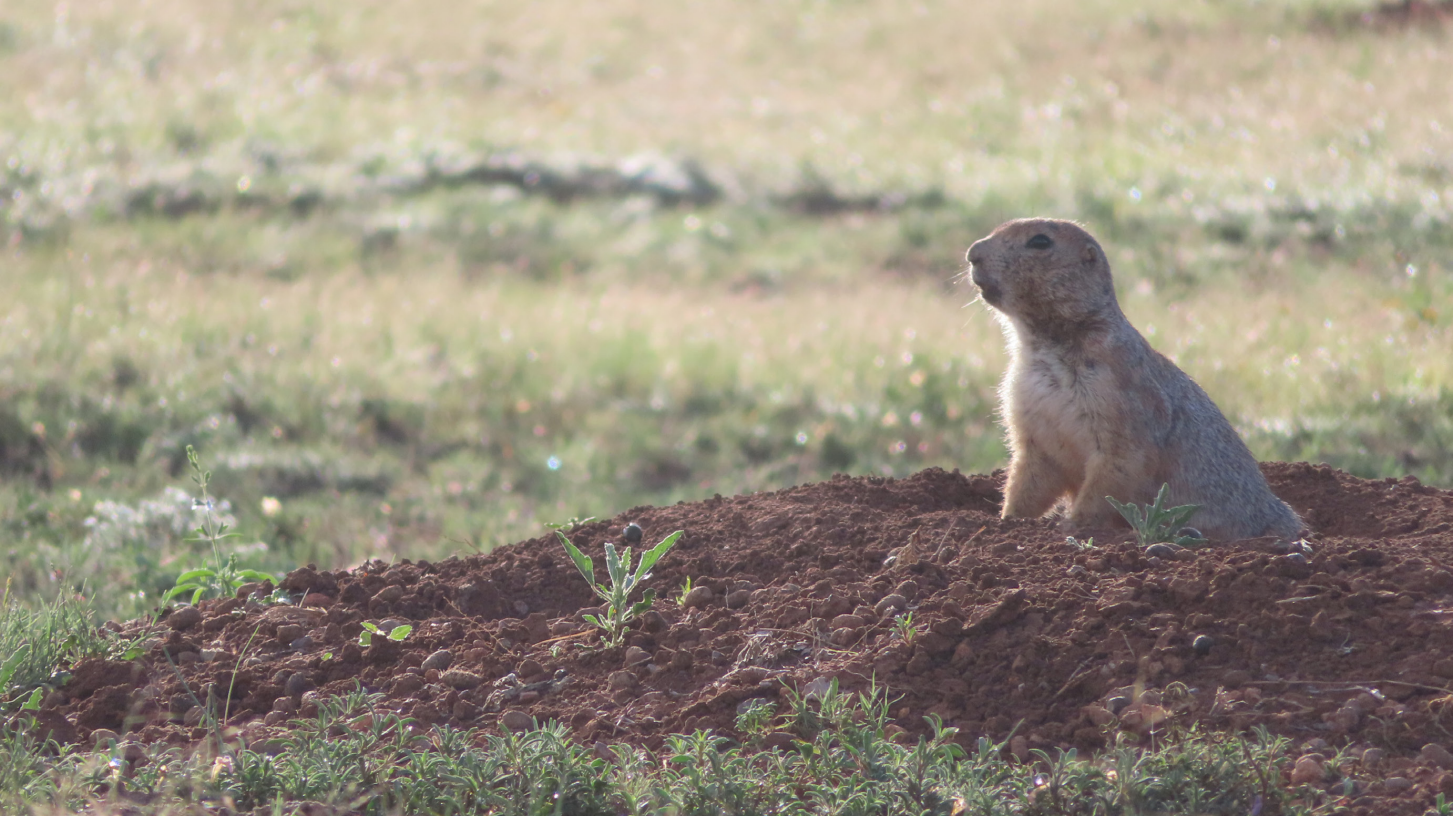
Prairie dog (*Cynomys mexicanus*) looking out of its burrow

Our aim here was to investigate vegetation responses (taxonomical and functional) to disturbance by the prairie dog species *Cynomys mexicanus* (endemic to northeastern Mexico; Figure 1) during the wet and dry season, throughout the different grassland types present in the Grassland Priority Conservation Area (GPCA) of El Tokio. We assume that functional diversity metrics will be more sensitive and will help to provide an in‐depth understanding of the mechanisms or patterns of community changes. Understanding these complex ecosystem interactions will help us understand the functional response of vegetation to prairie dog disturbance, which will aid future management and conservation strategies to protect both, prairie dogs and vegetation diversity to maintain the essential functions of semiarid grassland under future environmental changes. We therefore used the traits mentioned above and calculated plant functional diversity and CWM traits for the prairie dog‐dominated grasslands within GPCA El Tokio, to answer the following questions: (1) Is there an effect of prairie dog disturbance on taxonomical and functional plant diversity, and how are CWM traits being filtered? (2) Is the effect constant across different grassland types? (3) Does season influence these effects?

## 2 METHODS

### 2.1 Study area and species

**FIGURE 2:**
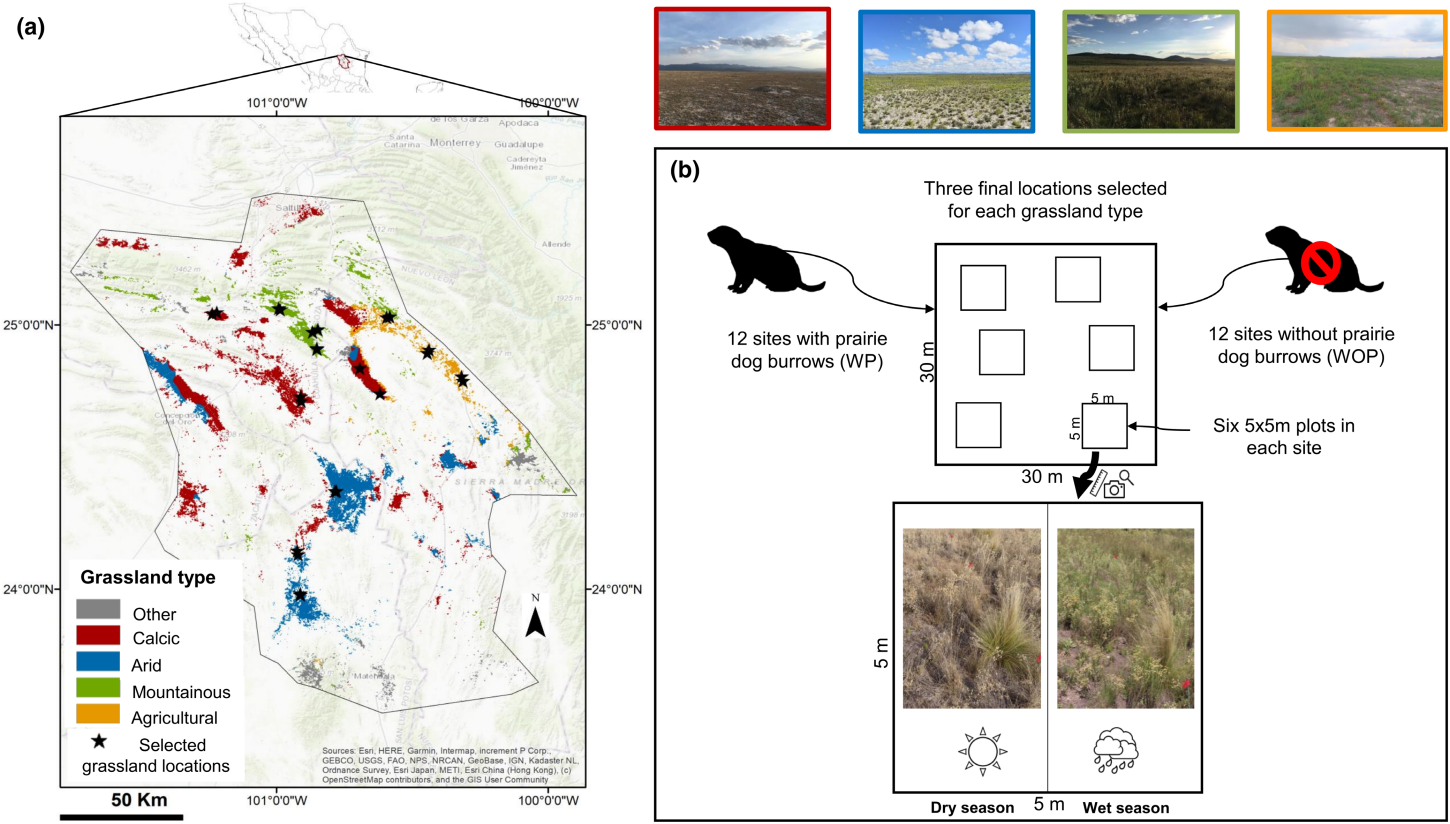
GPCA El Tokio study site in Mexico (encompasses the states of Nuevo Leon, San Luis Potosi, Zacatecas and Coahuila) and experimental design. (a) Grassland types and selected grassland locations (3 in each grassland type). (b) Experimental design: Each grassland location had one site with active prairie dog burrows (WP) and one site without (WOP). A 30 × 30 m quadrant was delimited in each site with 6 plots each, further divided into two temporal subplots

This study was conducted in the GPCA El Tokio (Figure 2a) within the Chihuahuan Desert in northeastern Mexico. El Tokio, designated as a GPCA by the Commission for Environmental Cooperation in 2009 due to its ecological importance and threatened nature (CEC, 2010), covers an area of 2.3 million ha and encompasses the Mexican states of Nuevo Leon, San Luis Potosi, Zacatecas and Coahuila. The area falls within the Meseta Central matorral ecoregion, considered as a Desert & Xeric Shrubland Biome (Dinerstein et al., 2017), except for the mountain grasslands within it, which fall within the Sierra Madre Oriental pine‐oak forests ecoregion. The climate of El Tokio is semiarid with mean annual temperatures between 16 and 18°C, a mean temperature of the driest quarter (January–March) of 13.9°C and a mean temperature of the wettest quarter (July to September) of 19.5°C (Baez‐Gonzalez et al., 2018). Precipitation ranges from 300 to 600 mm, with an average monthly precipitation of the driest quarter at 14.0 mm (January–March), here considered as the dry season, and an average monthly precipitation of the wettest quarter being 60 mm (July–September), here considered as wet season, (Baez‐Gonzalez et al., 2018). Altitude ranges from 1550 to 1800 m a.s.l., and the area has at least five different soil types, mostly gypsum and xerosol soils with low calcium carbonate content and a loamy‐silt texture, followed by loamy‐clayey soils and loamy‐sandy soils (Pando Moreno et al., 2013). The area consists mostly of natural halophyte and gypsophilous shrublands, with some remaining grasslands covering approximately 35,000 ha. These grasslands are today highly fragmented due to anthropogenic activities related to livestock and agriculture. Grassland vegetation is mostly dominated by the families Poaceae, Chenopodiaceae, and Frankeniaceae (Rzedowski, 2006). The dominant graminoid species are *Muhlenbergia villiflora* var. *villiflora*, *Scleropogon brevifolius*, and *Bouteloua dactyloides*. The region is also rich in endemic species such as *Nerisyrenia mexicana*, *Frankenia margaritae*, *Calylophus hartwegii* spp. *Maccartii*, and *Gaillardia comosa* (Estrada‐Castillón et al., 2010). Grasslands in the area tend to be of short‐grass nature and are characterized by discontinuous vegetation patches and high proportions of bare soil, which is common in dryland ecosystems (Valentin & Poesen, 1999). A limited number of studies have been carried out on plant competition in this habitat, but based on its halophytic‐gypsophilous soils and its semiarid characteristics, we can assume that water availability, herbivory and fertile soil patches play a key role (Blumenthal et al., 2020; Escudero et al., 2015). Adding more to the ecological significance of GPCA El Tokio is the fact that the area holds the last remaining colonies of *C. mexicanus*. The species is very similar in physical and behavioral characteristics to the better known *C. ludovicianus* (Castellanos‐Morales et al., 2016). It is considered a social species, and forms colonies that are composed of multiple burrows that can be up to 15 m long and are usually spaced out by several meters from each other (Whicker & Detling, 1988). Colonies can only be found in grasslands with little to no slope (no more than 8% inclination), with shortgrass and usually surrounded by vegetation with higher height (SEMARNAT, 2018).

### 2.2 Site selection and experimental design

#### 2.2.1 Data‐driven identification of grassland types

Based on *C. mexicanus* unique presence in grassland habitat, we identified all grasslands in the area based on available land use maps (NALCMS, 2017; Scott Morales & Vela Coiffier, 2017). To select a representative sample of sites covering the varying environmental conditions present in these grasslands, a data‐driven clustering approach was used. We used a self‐organizing map (SOM), a type of artificial neural network that is trained using competitive learning and well suited to finding clusters within data, as implemented in the package kohonen version 3.0.11 (Wehrens & Buydens, 2007; R version 4.0, R Core Team, 2020). Using geospatial environmental data (see Supporting Information S1 for the specific data sets used), this analysis clustered all grassland locations into eight groups, four of which occupy most of GPCA El Tokio and are therefore here considered as distinct grassland types (Figure 2a): (1) Agricultural (Agri): characterized by agricultural land use, xerosol haplic soils, total annual precipitation between 300 and 400 mm and temperature between 14 and 16°C; (2) Arid: characterized by solonchak orthic soils, low elevation, and total annual precipitation from 200 to 400 mm; (3) Calcareous (Calc): characterized by xerosol calcic soils, total annual precipitation between 300 and 400 mm, low elevation and temperatures between 14 and 16°C and (4) Mountain (Mount): characterized by litosol, high precipitation ranging from 400 to 500 mm, temperature between 14 and 16°C and high elevation.

#### 2.2.2 Study plots

First, a total of 49 independent grassland patches with active prairie dog colonies (from here on locations) were identified with the use of previous literature (Ceballos et al., 1993; Estrada‐Castillón et al., 2010; Scott‐Morales et al., 2004; Treviño‐Villarreal & Grant, 1998), up‐to‐date Google Earth Imagery and historical and present delimitations of colonies (provided by the Mexican organizations PROFAUNA and Organización Visa Silvestre A.C.‐OVIS). Three locations for each of the previously identified grassland types were selected, resulting in a total of 12 locations. Preference was given to locations where community‐based conservation projects had already been implemented or are currently implemented by local organizations to ensure feasibility of the study results for future conservation efforts within GPCA El Tokio. As a second filter locations had a spatial distance of at least 5 km between them, due to prairie dogs average dispersal distance (Garret & Franklin, 1988). Easy access to the locations was considered as a third filter. Previously, grazing by cattle had been documented in most locations (Estrada‐Castillón et al., 2010), but no detailed information was available on the number of cattle or stocking densities. Therefore, as a fourth filter, and to control for any differences between locations with cattle or no cattle grazing, we only selected locations where cattle activity was observed, or where fresh feces were found during field explorations. Once the 12 locations were chosen, the areas with active prairie dog burrows (WP) and without active prairie dog burrows (WOP) were delimited using Google Earth Imagery. In each delineated area, a 30 × 30 m random square was placed using ArcGIS. Squares were congruent to the cardinal directions. WP and WOP locations can be distinguished by the presence of prairie dog activity, feces, and burrows being not overgrown by vegetation, filled with dirt or covered by spider webs. Based on visual assessments, we found vegetation within the delimited WP and WOP locations to be mostly homogenous and generally consistent with descriptions of Estrada‐Castillón et al. (2010). However, we also ensured that the 30 × 30 m squares were representative, for example, did not fall on a road, or on a woody shrub patch. A total of 24 30 × 30 m squares (from here on sites) were selected. All WP and WOP sites had a minimum distance of 1 km, except for sites in one of the mountain locations, where WP and WOP sites were only 300 m apart due to lack of alternative areas. Selected sites in mountain, calcareous, and arid grasslands are within colonies that range from 12 to 6700 ha and have decreased in size since 2002 (between 12% and 77% loss; Table S2_1). Agricultural grasslands have been in constant land use change since 2002 (based on Google Earth imagery) and have been used for agriculture since 1950 (Treviño‐Villarreal & Grant, 1998). They are usually cultivated for 3–4 years and then abandoned for 5 years or more (Estrada‐Castillón et al., 2010). Using Esri World Imagery Map (Esri et al., 2021), we could identify the average burrow density which ranged from 0.89 to 3.71 depending on the location. Averages were obtained from multiple randomly selected 30 × 30 m squares (more information on Table S2_1). Within each site, six random 5 × 5 m plots, aligned along the sites edges. Randomization of these plots was performed by blindly throwing six 60 cm diameter rings to fall at random. In the case the ring or plot area overlapped, the rings would be thrown again. The burrow closest to the rings was selected as the center of the WP plots. Whenever the selected site had less than 6 burrows within it, all burrows were selected for plots and the leftover plots were randomly selected and assigned as non‐burrow plots. To account for seasonal effects, the 5 × 5 m plots were further halved to create two 5 × 2.5 m subplots, which from here on are considered as seasonal subplots. Seasonal subplots were assigned as eastern (rainy season) and western (dry season; Figure 2b) halves. Data collection took place during August–September 2019 (rainy season) and during December 2019–January 2020 (dry season).

### 2.3 Vegetation sampling and trait measurements

We compiled a full list of species, based on the list provided on Estrada‐Castillón et al. (2010), for each subplot. Plant cover for each species was estimated using a modified Daubenmire plot and its cover scale method (Daubenmire, 1968) where each species is individually assessed and classified within one of 6 designated cover classes and assigned a midpoint value: (1) 0%–5% = 2.5%; (2) 5%–25% = 15%; (3) 25%–50% = 37.50; (4) 50%–75% = 62.50%; (5) 75%–95% = 85%; (6) 95%–100% = 97.50%. A total of six traits were selected due to the feasibility to obtain them in the field (Reich, 2014) and their relationship with key grassland functions in vegetation studies (Garnier et al., 2007). Three traits, vegetative height (cm), leaf area (cm^2^), and habit, were assessed in the field following the guidelines by Pérez‐Harguindeguy et al. (2013). Vegetative height was measured for at least 2 healthy individuals per species, for each plot. Leaves were collected for at least 5 healthy individuals of each species within each location. Leaf area was measured within 3–5 h after collection using the app LeafByte, version 1.3.0. (Getman‐Pickering et al., 2020). Due to the COVID‐19 virus restrictions in Mexico, the measurement of leaf dry mass was not possible, so leaf area was used instead of SLA. Plant habit was considered as erect or prostrate to further specify the species life form. Traits obtained from the literature were life history (annual or perennial), photosynthetic pathway (C3 or C4), and life form (forb, graminoid, sub‐shrub, or shrub). Many of the plant species in GPCA El Tokio have been poorly studied; therefore, we did not use other traits. Furthermore, it was difficult to find information even for the traits commonly used in plant trait studies (Blumenthal et al., 2020). We could obtain traits for 63 of 92 of the species, which together accounted for 96% of the total cover.

### 2.4 Diversity metrics

#### 2.4.1 Taxonomic diversity metrics

Species richness and cover were averaged across the 6 seasonal subplots in each site using R version 4.0.3, as were all subsequent analyses. Species evenness was obtained by using the Inverse Simpson index (considered as “simpson”) in the “adiv” package version 2.1.1 (Pavoine, 2020). The index is calculated as follows: $\left(1/\sum \mathrm{jp}2\mathrm{ij}\right)/S_{i}$, where $S_{i}$ is the number of species in a community, pij is the relative abundance of species j in the community i. This index was selected due to its high sensitivity to both dominant and rare species with symmetry between them (Beisel et al., 2003; Smith & Wilson, 1996).

#### 2.4.2 Functional diversity metrics

Three functional metrics based on Villéger et al. (2008) and Mouillot et al. (2013) were selected: functional evenness (FEve), functional divergence (FDiv), and functional specialization (FSpe). These metrics were obtained by plotting all traits jointly in functional space and measuring the positions within this space in relation to the species abundances and trait distributions within it. To do this, we first calculated gower dissimilarities using daisy from the “cluster” package version 2.1.2 (Maechler et al., 2021). We also correlated gower dissimilarity matrices obtained by the cluster package and the gawdis package version 0.1.3 (de Bello et al., 2021), which is a type of weighted gower dissimilarity. We found both dissimilarity matrices were correlated and decided to keep the “cluster” gower distance, recommended by Villéger et al. (2008; Table S2_2). FEve measures the changes in abundance distributions within the functional space based on a Minimum spanning tree (MST); this metric indicates how abundances of species are distributed throughout the functional space, and it is higher when species abundances and species functional distance are similar. FDiv measures the changes in distance to the mean abundance (center) in relation to species abundances, that is, if species with high abundance have a greater distance than the overall mean, divergence will be higher. FSpe measures changes in abundance of generalist species (defined as species close to the center of the functional space) relative to the specialist species (species that have extreme trait combinations) by measuring the mean distance from the rest of the species pool in the functional space. Higher FSpe would indicate a higher community functional uniqueness relative to the pool of species present (Cornwell et al., 2006; Mouillot et al., 2013; Villéger et al., 2008). Functional richness (FRic) was not selected because it is highly correlated with taxonomic richness (Botta‐Dukát & Czúcz, 2016; Villéger et al., 2008). A fourth metric of functionality, Rao's quadratic entropy (RaoQ), was obtained with the “FD” package version 1.0.12 (Laliberté et al., 2015). The index follows the formula:

$$\text{RaoQ}=\sum_{I=L}^{S-1}\sum_{J=I+1}^{s}d_{\mathrm{ij}}p_{i}p_{i},$$

where $p_{i}$ is considered as *S*‐species community characterized by the relative abundance vector **p** = (*p*1, *p*2, …, *p*s) such that $\sum_{i=1}^{S}p_{i}=1$, and $d_{\mathrm{ij}}$ is the difference between the *i*‐th and *j*‐th species ($d_{\mathrm{ij}}$ = $d_{\mathrm{ji}}$ and $d_{\mathrm{ii}}$ = 0). RaoQ measures changes in the sum of weighted abundances of pairwise functions between species. It combines the information provided by FRic and FDiv and is suitable for detecting trait convergence and divergence. The higher the measure, the higher the dissimilarity and abundances of traits within the habitat (Botta‐Dukát & Czúcz, 2016). To obtain all previously mentioned indices of functional diversity, all numerical variables were standardized to zero mean and unit standard deviation to reduce the relative influence of variables in different orders of magnitude prior to analysis. To examine the overall differences between individual traits, we also obtained CWM using the “FD” package (Laliberté et al., 2015).

### 2.5 Statistical analysis

As an exploratory analysis to identify dissimilarities in composition of species, we identified unique species between WOP and WP and grassland type gamma diversity. Furthermore, a Correspondence Analysis (CA) was used for all grassland types together and for each grassland type independently using the “vegan” package, version 2.5‐7 (Oskanen et al., 2020). We chose this method because it is a great tool to simplify tables, and identify patterns of the relative composition without emphasizing differences in abundant species (David, 2017). To further test how grassland types, prairie dog grazing and seasons relate to taxonomic, functional trait and CWM measures, generalized and linear mixed models were fitted. Prairie dog disturbance (WP and WOP), season (wet or dry), and grassland type (Agri, Arid, Mount and Calc) were treated as fixed factors and grassland location names as a random factor to account for the variability between locations. Residuals were used to examine normality and homoscedasticity. Most response variables were transformed to achieve a normal distribution. Linear mixed models were fitted using the lme4 package version 1.1.27.1 (Bates et al., 2015); RaoQ, CWMheight, CWMleaf area, C3 cover were log transformed, whilst for annual cover, prostrate cover, forb cover, sub‐shrub cover +1 was added before they were log transformed. Species richness was not transformed, and was analyzed using a generalized linear mixed model following a Poisson distribution. FEve, FDiv, Fspe, and evenness range between zero and one; therefore, they were analyzed using the glmmTMB package version 1.1.2.3 with a beta distribution (Brooks et al., 2017). Degrees of freedom, *F*‐tests and *χ* ^2^ for glmms and lmms were obtained using parametric bootstrap with 10,000 iterations and the Kenward–Roger's approximation, respectively. Both methods were obtained from the pbkrtest package version 0.5.1 (Halekoh & Højsgaard, 2014). For models that followed the beta distribution, ANOVA tables from the car package (Fox & Weisberg, 2019) were used with type II sums of squares whenever there was no interaction, and type III sums of squares when there was an interaction. We considered all the predictor variables and their interactions to be biologically important and hence included them all in the full model. Best fit models were chosen based on multi‐model inference using dredge from the MuMIn package version 1.42.1 by comparing AICc (Bartoń et al., 2018; Table S2_3) and selecting the model with the lowest one. Once the best fit model was selected, Tukey's HSD post‐hoc test was used to compare levels within variables using the emmeans package version 1.5.4 (Lenth, 2021) and marginal pseudo‐*R* ^2^ ($R_{m}^{2}$) values were obtained with the Nakagawa et al. (2017) method available in the performance package version 0.8.0 (Lüdecke et al., 2021). Error probabilities (*p*‐values) are interpreted as recommended by Muff et al. (2021) with respect to their strength of evidence rather than significance, with the following suggested ranges: (1) 1 to 0.1 = little or no evidence; (2) 0.1 to 0.05 = Weak evidence; (3) 0.05 to 0.01 = Moderate evidence; (4) 0.01 to 0.001 = Strong evidence and (5) 0.001 to 0.0001 = Very strong evidence.

## 3 RESULTS

### 3.1 Effects on composition and diversity measures

**FIGURE 3:**
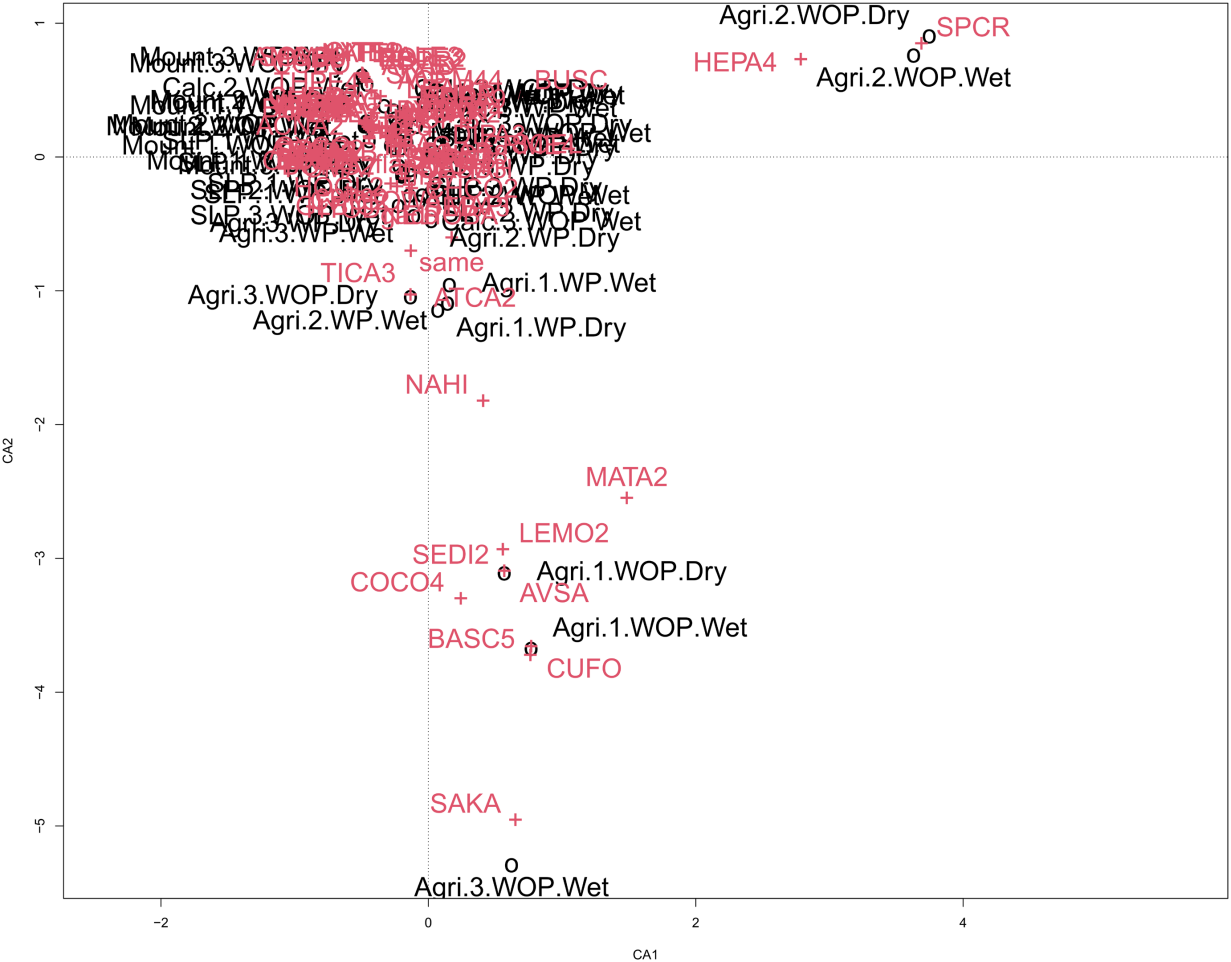
Correspondence analysis (CA) for all grassland types based on species abundances and sites. Species names are shown in red and sites names are shown in black. Eigenvalue/proportion explained: CA1 = 0.86/9.2%, CA2 = 0.83/8.8%. Species symbols can be found in Table S2_4

A total of 92 species were recorded (Table S2_4). There were no clear dissimilarity patterns of composition between WOP and WP in any of the grassland types, except for agricultural sites, where the mayor species contributing to dissimilarity were *Kochia scoparia*, *Cucurbita foetidisima*, *Conyza coulteri*, *Selenia dissecta*, *Sporobolus cryptandrus*, and *Heliopsis*. *Parvifolia* species. These species appeared either only, or in some cases, less frequently in WP than in WOP sites (Figure 3), results on individual grassland types showed no clear dissimilarity patterns between WOP and WP (Figure S2_1).

**FIGURE 4:**
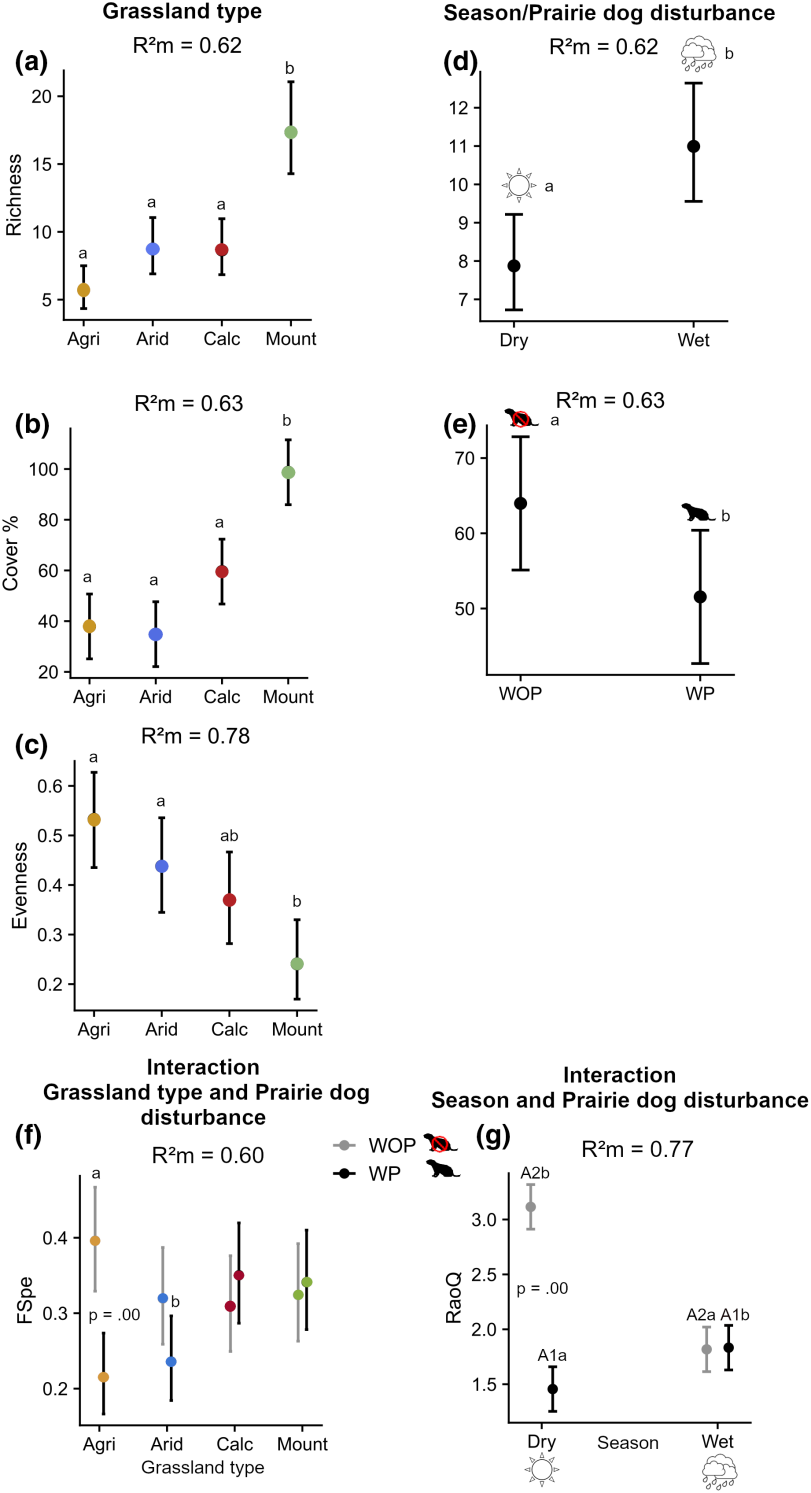
Comparison of marginal effects on different diversity indices (both taxonomic and functional). Effects are shown only for fixed effect estimates of uncertainty. The graphs are shown only for models revealing very strong, strong and moderate evidence of effects. For models with no interaction effects with disturbance (a–e): Results with *p* < .05 are represented by lowercase letters, levels sharing a letter have no evidence of being affected. For models with interactions (f–g): (f) FSpe: Results comparing prairie dog disturbance in the same grassland type are shown with *p* < .05, at least moderate evidence of effects between grassland types are indicated by lowercase letters. (g) RaoQ: Differences between prairie dog disturbance in the same season are shown with *p* < .05. Difference of WP between seasons is represented by A1; difference of WOP between seasons is represented by A2. Differences between WOP‐dry and WP‐wet are represented by b; differences between WP‐dry and WOP‐wet are represented by a

Overall, models of measures related to species diversity, that is, richness, evenness, and cover showed no evidence of interaction effects with disturbance, but all measures were influenced by grassland type. Species richness was influenced by season and there was only moderate evidence of cover being influenced negatively by prairie dog disturbance. Mountain grasslands had the highest richness and cover compared to all other grassland types, but had the lowest evenness. The wet season positively affected richness compared to the dry season. There was moderate evidence of prairie dog disturbance having interactive effects on functional diversity measures, specifically on FSpe and RaoQ (Table 1; Figure 4). There was strong evidence of grassland type moderating the effect of prairie dog disturbance on FSpe. This effect was particularly important for agricultural grasslands where sites with prairie dogs had a lower FSpe than sites without prairie dog disturbance (Figure 4f). RaoQ showed strong evidence of being influenced by the interaction of prairie dog disturbance and season. Grasslands without prairie dog disturbance (WOP) had higher RaoQ during the dry season compared with the wet season. There was no evidence of differences in RaoQ between seasons for grasslands disturbed by prairie dogs (WP). In the wet season, no evidence was found regarding differences between conditions of prairie dog disturbance, in contrast to the dry season where RaoQ values varied greatly between conditions. This indicates that prairie dog disturbance did not cause functionally unique communities. Moreover, communities without the disturbance where unique only in agricultural grasslands, and only during the wet season in the case of RaoQ. Furthermore, trait values seem to be similar in all communities, as indicated by the lack of evidence that FEve was influenced by any of the variables nor their treatments or interactions. This was also the case for Fdiv, which showed no evidence after pairwise post‐hoc analysis (Table S2_5).

**TABLE 1:** Results of linear and generalized linear mixed models to test how grassland types, prairie dog grazing and seasons relate to taxonomic, functional and CWM trait measures. Prairie dog disturbance (WP and WOP), season (wet or dry) and grassland type (Agri, arid, mount and calc) were treated as fixed factors and grassland location as a random factor. The table is shown only for final models selected based on Akaike's information criterion for small samples (AICc)

Explanatory variables	nDF	dDF	Test	*p* value	Figures
Diversity measures					
Richness			*χ* ^2^		
Grassland type	3	—	19.23	.00	Figure 4a
Season	1	—	13.62	.00	Figure 4d
Cover			*F*‐test		
Grassland type	3	8	21.53	.00	Figure 4b
Prairie dog disturbance	1	35	4.182	.05	Figure 4e
Evenness			*χ* ^2^		
Grassland type	3	—	20.23	.00	Figure 4c
FSpe			*χ* ^2^		
Grassland type	3	—	4.35	.23	
Prairie dog disturbance	1	—	32.30	.00	
Grassland type × Prairie dog disturbance	3	—	31.68	.00	Figure 4f
RaoQ			*F*‐test		
Prairie dog disturbance	1	34	26.50		
Season	1	34	8.31		
Prairie dog disturbance × Season	1	33	81.69	.00	Figure 4g
Traits					
Perennial cover			*F*‐test		
Grassland type	3	8	8.00	.01	Figure 5a
Erect cover			*F*‐test		
Grassland type	3	8	13.51	.00	Figure 5b
Prairie dog disturbance	1	35	3.53	.07	
Graminoid cover			*F*‐test		
Grassland type	3	8	10.85	.01	Figure 5c
Prairie dog disturbance	1	34	2.77	.11	
Season	1	34	1.61	.21	
Prairie dog disturbance × Season	1	33	4.35	.05	Figure 5i
C4 cover			*F*‐test		
Grassland type	3	8	5.66	.03	Figure 5d
Prairie dog disturbance	1	34	5.77	.02	Figure 5h
Annual cover			*F*‐test		
Season	1	34	15.68	.00	Figure 5e
Forb cover			*F*‐test		
Season	1	34	12.34	.00	Figure 5f
CWM Leaf area cover			*F*‐test		
Prairie dog disturbance	1	34	3.04	.09	
Season	1	34	6.66	.01	Figure 5g

*Note*: The table shows the test type *χ* ^2^ and *F*‐test. nDF, numerator degrees of freedom; dDF0, denominator degrees of freedom; Emmeans test *p*‐adjust, Tukey, comparisons of levels within the variables from Tukey's HSD post‐hoc test that show weak to very strong evidence of having an effect.

### 3.2 Trait filtering effects

**FIGURE 5:**
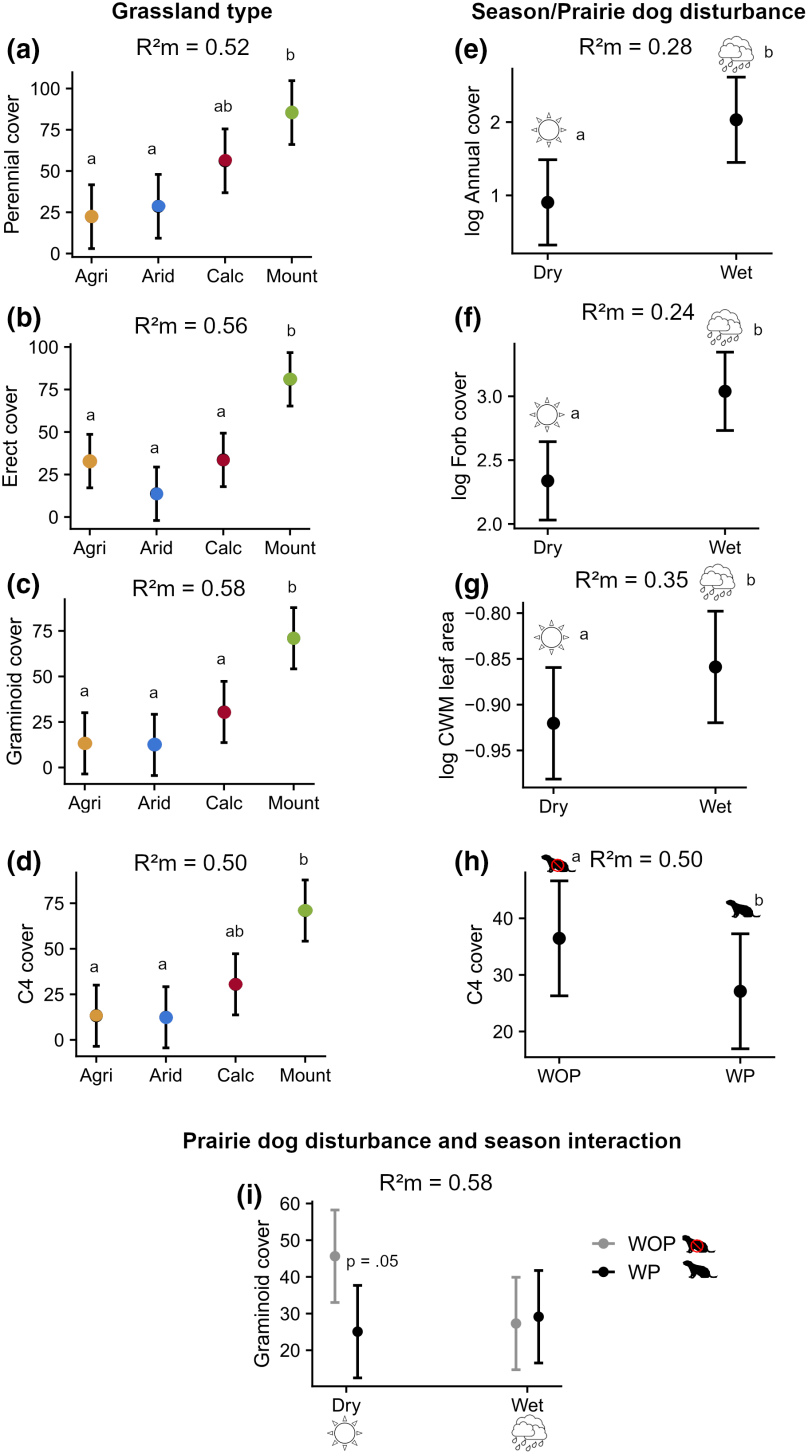
Comparison of marginal effects on trait filtering model effects. Effects are shown only for fixed effect estimates of uncertainty. The graphs are shown only for models revealing very strong, strong, and moderate evidence of effects. For models with no interactions (a–h): Results with *p* < .05 are represented by lowercase letters, levels sharing a letter had weak to no evidence of effects. For models with interactions: (i) graminoid cover: Results comparing prairie dog disturbance in the same season are shown with *p* < .05

Effects of prairie dog disturbance were captured only by C4 cover and graminoid cover, whereby graminoid cover was mediated by an interaction with season and C4 differences were explained by grassland type and prairie dog disturbance but not by an interactive effect. Grassland type had an effect on almost all traits except for annual cover, forb cover and leaf area which were influenced mostly by season (Table 1; Figure 5). There was strong evidence of mountain grasslands having the highest cover, compared to arid and agricultural grassland types in perennial cover, and to all other grassland types in erect and graminoid cover. There was weak evidence of prostrate cover being higher in calcic grasslands compared with agricultural sites (Table S2_5). Annual cover, forb cover, and CWM leaf area revealed strong evidence of increasing during the wet season. There was only weak evidence of C3 species cover having a higher response to the wet season (Table S2_5). There was moderate evidence of C4 cover being higher in mountain grassland and compared to agricultural and arid grassland types which had a lower C4 cover and higher in WOP sites. There was no evidence of graminoid cover having differences between seasons for WP sites. On the contrary, WOP sites showed contrasting effects between seasons, having almost double graminoid cover during the dry season, revealing moderate evidence of a positive effect compared to the WP sites. There was no evidence of CWM vegetation height having an effect on any of the grassland types, sites or seasons conditions (Table S2_5).

## 4 DISCUSSION

### 4.1 Effects of prairie dog disturbance on diversity and CWM means

Disturbance by prairie dogs has been shown to affect multiple vegetation parameters (Connell et al., 2019; Duchardt et al., 2021). Therefore, the main objective of this study was to investigate the effect prairie dogs have on the GPCA El Tokio grasslands, using functional and taxonomic diversity measures. In contrast to studies stating that prairie dog disturbance has negative effects on cattle feed efficiency, for example, forage consumption (Derner et al., 2006; Vermeire et al., 2004), we found only moderate evidence of higher cover in WOP sites. Furthermore, the majority of taxonomical and functional metrics tested in our study were not affected by disturbance, and instead were mostly controlled by grassland type and season. This results indicate that environmental variables play a stronger role than grazing and animal disturbances on vegetation in shortgrass‐dominant grasslands and is in line with studies in similar ecosystems (Grinath et al., 2019; Jäschke et al., 2020; Török et al., 2018). Moreover, this strong environmental effect on vegetation is in line with a recent study from Augustine and Derner (2021), which suggested prairie dog disturbance did not impact cattle mass gain negatively due to the influence of topography, temporal and soil variability. It is also important to note that prairie dog disturbance had no effects on the CWM height and leaf area, traits that are usually associated with grazing pressure (Blumenthal et al., 2020; Díaz et al., 2006). In fact, CWM leaf area was only dependent on season and there was no evidence that CWM height was affected. We found moderate to weak evidence that prairie dog disturbance did filter C4 cover, which was higher in sites without prairie dog disturbances. This can be explained by the fact that prairie dogs prefer to feed on grasses (Mellado et al., 2005). Most grasses present in the study area are C4, specifically the grasses with highest cover such as *Sporobolus cryptandrus* in agricultural grasslands, *Aristida pansa* in calcareous grasslands and *Bouteloua dactyloides* in mountain grasslands, and so a lower cover would be expected. However, a recent study, covering a period of 72 years (Augustine et al., 2017), showed that some of these C4 species are being outcompeted by C3 species in the long term, especially in the absence of grazing.In addition, we found that functional diversity, but not species diversity, responded to the joint effects of grassland type and seasonality with prairie dog disturbance, confirming not only the need of including multiple environmental variables and their interactions to identify ecosystem complexity (Dainese et al., 2015), but also the importance of considering functional diversity to further understand the instances of these patterns (Cadotte et al., 2011). Prairie dog disturbance moderated FSpe in agricultural grasslands, possibly explained by the suppression of rapid growing species with extreme traits (e.g., *Salsola kali*, *Machaeranthera tanacetifolia*, and *Kochia scoparia*) that have higher LA and height and are able to grow and dominate in agricultural grasslands without prairie dog disturbance; which was also corroborated by the high dissimilarity of these species in the Correspondence Analysis. These species grow despite the lack of ideal water availability and soil conditions, because they benefit from the gain of resources due to nutrients from fertilization that remain after abandonment (Laliberté et al., 2012). Prairie dogs need short vegetation for predator avoidance (Hoogland, 1995), and their suppressing effect has been shown in previous literature (Hale et al., 2020; Ponce‐Guevara et al., 2016). However, as the mechanism behind this suppression is unclear, further studies are needed to determine whether prairie dogs colonize agricultural grasslands before or after rapid‐growing species have a chance to grow, or whether other mechanisms are at work (e.g., drought avoidance; Blumenthal et al., 2020). We further found no evidence of positive or negative effects on FEve and FDiv between grassland types, nor between any other of the measured conditions. This indicates that traits were mostly unchanged in their distribution and abundance between communities in the functional space volume. Our results are in accordance with the studies of Carmona et al. (2012) and Jäschke et al. (2020), showing that these plant traits are usually unaffected by grazing under restrictive water availability conditions. Therefore, we can assume that the redundancy of traits is most likely increasing due to the restrictive environmental conditions, such as gypsum soil and low precipitation in GPCA El Tokio, which only well‐adapted species can withstand (Mouillot et al., 2013; Villéger et al., 2008). Likewise, the restrictive environmental conditions might also explain the lack of clear composition dissimilarity patterns between WOP and WP by the correspondence analysis (Figure 3; Figure S2_1).

It is also important to mention that other aspects not fully considered in our study could positively influence the minor effects we found of prairie dog disturbance on vegetation functional and taxonomical diversity. For example, we here focus on grassland ecosystems. We found that WP sites contribute to the overall landscape gamma diversity (Table S2_4). Yet, WP sites have lower species richness, and a lower number of unique species than WOP sites within all grassland types. However, other studies (e.g., Baker et al., 2012) have shown the strong positive effects prairie dogs have on overall landscape diversity when considering both shrub and grassland ecosystems. Furthermore, prairie dogs have been shown to increase multiple ecosystem functions, such as soil productive potential and water infiltration, which could have direct or indirect effects on vegetation, but was not considered for this study (Martinez‐Estevez et al., 2013).

### 4.2 Grassland types as important effect drivers

We found that grassland types and not prairie dog disturbance explained most of the effects on plant functional and taxonomic diversity as well as CWM of traits. There was very strong evidence that mountain grasslands were positively affected in almost all measures, usually followed by arid, calcareous and agricultural grasslands, respectively. This can be explained by the tendency of mountain grasslands to have leptosol soils, highly variable slopes as well as higher elevation and lower atmospheric pressure, leading to higher precipitation and lower temperatures (Anjos et al., 2015; Gommes, 2002). These conditions are known to often cause an increase in plant species richness and cover (Buzhdygan et al., 2020; Speed et al., 2013). In addition, Pando Moreno et al. (2013) found that many of the sites in mountain grasslands within GPCA El Tokio had a lower level of electrical conductivity and absence of gypsum, whilst sites that fall within calcareous, arid and agricultural grassland types had at least some percentage of gypsum in them. Gypsum soils are known to limit plant life due to their chemical and physical properties which restrict plant growth (Escudero et al., 2015), it is therefore likely that the presence of gypsum acts as a habitat filter for CWM traits. Calcareous and mountain grasslands had similar filtering effects on perennial and C4 cover. The effect could be explained because calcareous soils have lower gypsum levels; and higher precipitation compared to the arid grasslands, therefore being less restrictive for vegetation, and having a similar response as mountainous grasslands for vegetation. Arid grasslands in this study are also dominated by gypsum soils and have higher temperatures which, together with low precipitation, result in higher level of aridity which can act as a strong filter for most CWM traits (Munson et al., 2013; Vicente‐Serrano et al., 2012). Similarly, the strong filtering effect of agricultural grasslands is most likely due to the land use history of agricultural grasslands, which allows the establishment of new, less adapted, species (Gustavsson et al., 2007), opposed to the restrictive conditions faced by vegetation on gypsum and calcareous soils (Meyer et al., 1992). Future studies, disentangling the climatic and edaphic effects of these grasslands types are needed to properly understand patterns of their interactive effect on vegetation (Le Bagousse‐Pinguet et al., 2017). On the other hand, the Inverse Simpson evenness showed an opposite result compared to the other diversity metrics for which grassland type had a strong effect. It was higher for agricultural grasslands, which may be explained by the fact that the index assigns a higher evenness value to communities with an almost equal amount of rare and dominant species (Smith & Wilson, 1996). Hence, the higher the number of rare species is, the lower is the Inverse Simpson evenness (Magurran, 2004).

### 4.3 Seasonal effects

Season affected species richness, cover of species with annual life history, forb growth form, and CWM leaf area independently to prairie dog disturbance. Leaf area and other leaf traits are considered to be directly related to the amount of water plants receive, especially in dry habitats (Sack & Holbrook, 2006; Wellstein et al., 2017). Most plants thus have higher leaf area during the wet season. Additionally, multiple studies have shown that annual and forb species strongly respond to increased precipitation levels (Spence et al., 2016; Yan et al., 2015). This is most likely due to their high germination rates and seed innate and water‐controlled dormancy, as well as specific dispersal adaptations (Freas & Kemp, 1983; Miranda et al., 2009) which together allow them to grow when the best conditions occur. In line with other studies from shortgrass and arid environments, our study shows that seasonality plays a bigger role on annual plant species and forb cover than prairie dogs. A result which has shown to be different in mixed‐grass prairie habitats where precipitation most likely does not play such a big role (Baker et al., 2012; Pérez‐Camacho et al., 2012). Furthermore, we think the lack of evidence on the positive effect of prairie dogs on annual cover increases (Augustine et al., 2014), could be explained by the specialized soil types which allow only for certain species to be present, and the presence of *Muhlenbergia villiflora* on all grassland types. However, understanding these dynamics was beyond the scope of this study and further research is needed to unravel these relationships.

Moreover, season modulated the effect of prairie dog disturbance on RaoQ. We found strong evidence that this index was different between prairie dog disturbance conditions during the dry season, where disturbed sites had a lower RaoQ. No difference, however, was found between disturbance conditions during the wet season. Interestingly, this result is consistent with a recent 3‐year study conducted in the highly distinct mixed‐grass prairies of northeastern Wyoming (Connell et al., 2019). The similar results could be due to the influence of WOP mountain grasslands in GPCA El Tokio, which have taller vegetation compared to the vegetation in all other grassland types (Table S2_6). Furthermore, we found no differences in graminoid cover for sites with prairie dog disturbance between the dry and the wet season. Additionally, there was strong evidence that sites without prairie dog disturbances increased graminoid cover during the dry season. This is most likely due to prairie dogs feeding on graminoids after the wet season, which reduces the grass cover that could remain in the dry season but allows to maintain an overall stable graminoid cover throughout the year (Mellado et al., 2005). However, due to the nature of drylands to have variable precipitation (D'Odorico & Bhattachan, 2012), further long‐term studies are needed to monitor these interactions, especially in light of future climate change projections for the area, which predict an increase in rainfall variability (Baez‐Gonzalez et al., 2018). Likewise, although our results show interactions between seasonality and disturbance, these effects only show short‐term trends. Sampling multiple years and seasons is necessary to obtain an overall pattern and identify the mechanisms behind it, as so many variables are interdependent and most likely have non‐linear effects (Paruelo et al., 2008).

## 5 CONCLUSION

To the best of our knowledge, this is the first study to examine the effects of prairie dog disturbance on vegetation using functional diversity metrics. Like previous research, our findings support the idea that community trait‐based measures are closely associated with abiotic (grassland types and season) and biotic (prairie dog disturbance) filtering, compared with taxonomy‐based approaches. Tailored management strategies using vegetation traits as a proxy to understand vegetation responses to environmental pressures will be key for the conservation and restoration of this threatened, semiarid ecosystem. The use of traits can provide information on how and to what extend is vegetation being most affected by the environment, helping managers to focus efforts on the traits that are being most impacted. Additionally, we found that prairie dogs had only a minor negative effect on vegetation cover, even though our study design focused on burrows and surrounding disturbance, favoring the detection of stronger differences between conditions with and without active prairie dog colonies. The effects of prairie dogs on C4 and graminoid cover were particularly demonstrated in the dry season, with the latter negatively affecting functional diversity only in the dry season, while offsetting it in the wet season. Our study provides further evidence of the large impact environmental conditions have on these short‐grass, water‐restricted grassland ecosystem. It is therefore likely that plant responses will be negatively affected under future climate change scenarios. Hence, longer‐term interannual variation studies combining both types of diversity measures should be undertaken. Future studies in GPCA El Tokio can take advantage of the fixed location of prairie dog disturbance, as well as varying environmental conditions within the relatively small area, to assess responses of different grasslands to disturbance and environmental change.

## AUTHOR CONTRIBUTIONS

**Maria Gabriela Rodriguez‐Barrera:** Conceptualization (equal); data curation (lead); formal analysis (lead); funding acquisition (lead); investigation (lead); methodology (lead); project administration (equal); writing – original draft (lead); writing – review and editing (lead). **Ingolf Kühn:** Conceptualization (supporting); formal analysis (supporting); investigation (supporting); supervision (supporting); writing – review and editing (supporting). **Andrés Eduardo Estrada Castillón:** Data curation (supporting); writing – review and editing (supporting). **Anna Cord:** Conceptualization (equal); formal analysis (supporting); funding acquisition (supporting); investigation (supporting); methodology (supporting); project administration (supporting); supervision (lead); writing – review and editing (equal).

## CONFLICT OF INTEREST

The authors declare no conflict of interest.

## Supporting information


Appendix S1

Appendix S2

## Data Availability

Plant trait data available in the Dryad Digital Repository https://doi.org/10.5061/dryad.h9w0vt4mc (Rodriguez Barrera et al., 2022).
