# Supplementary material for: Grassland type and seasonal effects have a bigger influence on plant functional and taxonomical diversity than prairie dog disturbances in semiarid grasslands
Source: Ecol Evol. 2022 Jul 13;12(7):e9040. doi: 10.1002/ece3.9040 (PMC9279056; doi:10.1002/ece3.9040)
Supplement: Supplementary file 1 — Appendix S1 [file ECE3-12-e9040-s001.pdf]

A. ENVIRONMENTAL VARIABLES

Study area grasslands grid

The limits of GPCA El Tokio were obtained from the Commission for Environmental Cooperation (2010). Identification of grasslands was based on prairie dog presence points from Scott-Morales et al. (2016) and grassland features obtained from land use layers. Features related to grasslands were extracted from CONABIO 30m land use map from 2010 (Selected by attributes "legend" (Value 7, Matorral tropical o subtropical; Value 9, Pastizal tropical o subtropical; Value 10, Pastizal templado o subpolar; Value 15, Suelo agrícola and Value 16, Suelo desnudo)) and the INEGI vi 1:250000 (Selected by attributes "legend" (PY: Pastizal Gipsolfilo; PH: Patizal Halofilo; PN: Pastizal Natural; PI: Pastizal Inducido)). The resulting shapefiles were merged and ground truthed with Google Earth imagery.

Majority Slope

Slope was calculated from the 30m DEM- ASTER GDEM Version 2 (ASTGTM V002) (Slope: Output measurement = Degree). The "majority" resampling technique was used to determine the new value of a cell based on the most popular values of a filter window.

Majority soil type (edaphology)

The 1:1000000 shapefile with soil classifications was converted to a 30m raster based on the dominant soil type. The "majority" resampling technique was used to obtain a new value based on the most popular values of a filter window. The resulting raster values are shown in table 1.

Table 1. Description of soil types as given from World Reference Base for Soil Resources (WRB) from 1998, 2006, and 2015.

Table 1. Edaphology classifications

Raster value	Name	Texture	Physical phase	Description
1	XEROSOL CALCIC	fine	Petrocalcic	Soild with a white layer, rich in lime, and in a form of white powder or caliche. Fertility of this soils go from moderate to high

2	LITOSOL	medium	Petrocalcic	Gravel soils on top of continuous rock. When over calcareous rock these are usually considered as Rendzinas.
3	XEROSOL HAPLIC	Medium	Petrogypsic	Soils that do not present characteristics of other existent subunits in certain types of soil.
4	FEOZEM CALCARIC	medium	gravel	Calcaric between 20 and 50 cm from surface
5	REGOSOL CALCARIC	medium	na	Soils that do not present characteristics of other existent subunits in certain types of soil.
6	RENDZINA	medium	Petrocalcic	Unclassified soils. Uusually mineral soils not yet developed. Extended on highly eroded soils.
7	KASTANOZEMS HAPLIC	medium	Petrocalcic	Dark brown soils rich in organic matter
8	KASTANOZEMS CALCIC	medium	Petrocalcic	Dark brown soils rich in organic matter
9	SOLONCHAK ORTIC	medium	Petrogypsic	High on soluble salts. Confined to arid, semiarid and costal areas.
10	XEROSOL GYPSIC	medium	na	Soild from arid or semi-arid zones that present acumulated gypsum (plaster) in crystal form. This layers can be ligh pink
11	KASTANOZEMS LUVIC	medium	Petrocalcic shallow	Dark brown soils rich in organic matter
12	FEOZEM LUVIC	medium	gravel	Presence of an argic horizon, high in white clay.
13	XEROSOL LUVIC	medium	gravel	Soild with clay accumulation in their subsoil. Generally color red.
14	na	na	na	na
15	CAMBISOL CALCIC	medium	na	Soils with at least an incipient subsurface soil formation. Transformation of parent material is evident from structure formation and mostly brownish discoloration, increasing clay percentage, and/or carbonate removal

DEM

The multiple 30m DEM layers from locations N23W101, N23W102, N24W100, N24W101, N24W102, N25W101, N25W102 were joined together using the mosaic method. All layers were obtained from ASTER GDEM Version 2 (ASTGTM V002), a product of Japan's Ministry of Economy, Trade, and Industry (METI) and NASA.

Annual temperature

The 1:1000000 shapefile was converted to a 30m resolution raster based on temperature ranges. Raster values were: (1) 14-16 °C (2) 16-18 °C (3) 12-14°C (4) 10-12 °C (5) 18-20 °C.

Annual total precipitation

The 1:1000000 shapefile was converted to a 30m resolution raster based on the precipitation ranges. The applied raster values were: (1) has a range from 200 to 300mm, (2) 300 to 400mm (3) 500 to 600mm (4) 400 to 500 mm.

Table 2. Selected environmental variables. Timespan, scale, type and reference are provided.

Variable	Time (span)	Type	Scale	References
ASTER GDEM Version 2 (ASTGTM V002)	2011	Raster	30 m	ASTER GDEM is a product of Japan's Ministry of Economy, Trade, and Industry (METI) and NASA."
Edaphology (Majority soil class)	1995	Vector	1:1000000	Instituto Nacional de investigaciones Forestales y Agropecuarias (INIFAP) - Comisión Nacional para el Conocimiento y Uso de la Biodiversidad (CONABIO), (1995). "Mapa edafológico". Escalas 1:250 000 y 1:1 000 000. México.
Total annual precipitation	1998	Vector	1: 1000000	García, E. - CONABIO, (1998). 'Precipitación total anual'. Escala 1: 1000000. México.
Mean annual temperature	1910-2009	Vector	1: 1000000	Cuervo-Robayo, A. P., Téllez-Valdés, O., Gómez-Albores, M. A., Venegas-Barrera, C. S., Manjarrez, J., Martínez-Meyer, E., (2014). 'Temperatura media anual en México (1910-2009)', escala: 1:1000000. Modified by CONABIO (2015). México, D. F..
Slope	2011	Raster	30m	Calculated from 30m DEM raster

References:

Comission for Environmental Cooperation (CEC). 2010. Priority Conservation Areas: Grasslands, 2010. Vector digital data.

Cuervo-Robayo, A. P., Téllez-Valdés, O., Gómez-Albores, M. A., Venegas-Barrera, C. S., Manjarrez, J., Martínez-Meyer, E., (2014)

FAO: World Reference Base for Soil Resources, by ISSS–ISRIC–FAO. World Soil Resources Reports 84. FAO, Rome 1998.

García, E. - CONABIO, (1998). Precipitación total annual.

Instituto Nacional de investigaciones Forestales y Agropecuarias (INIFAP) - Comisión Nacional para el Conocimiento y Uso de la Biodiversidad (CONABIO), (1995). 'Edafología'. Escalas 1:250000 y 1:1000000. México.

IUSS Working Group WRB: World Reference Base for Soil Resources 2006. World Soil Resources Reports 103. FAO, Rome 2006.

IUSS Working Group WRB: World Reference Base for Soil Resources 2014, Update 2015. World Soil Resources Reports 106, FAO, Rome 2015

Scott-Morales, L. M., P. Vela-Coiffier, (2016). 'Cynomys mexicanus (perrito llanero mexicano). Registros de presencia usados para elaborar el mapa de distribución potencial', edición: 1. Universidad Autónoma de Nuevo León e Instituto Tecnológico y de Estudios Superiores de Monterrey. Proyecto: JM004, Modelos predictivos de distribución geográfica para Spizella wortheni, Cynomys mexicanus y Taxidea taxus berlandieri y Vulpes macrotis. CONABIO. Monterrey, Nuevo León, México.

B. DETERMINATION OF CLUSTER NUMBER

A)

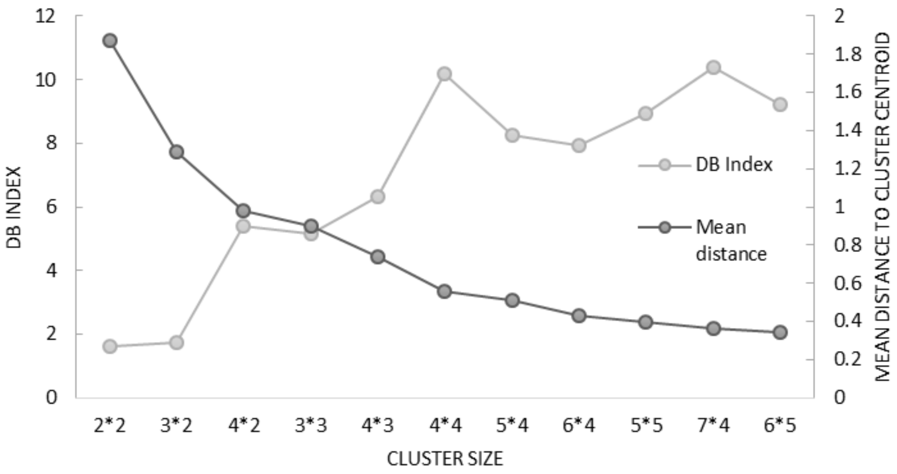

SOM neighbour distances

B)

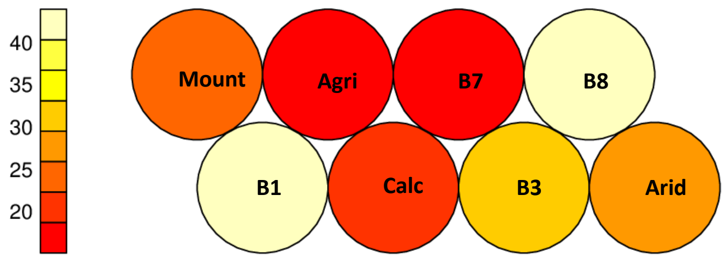

Figure 1: We tested multiple cluster numbers (four to thirty) by selecting differently shaped output planes. Two analysis were used to define sensitivity of clusters. One sensitivity analysis is the Davies Bouldin index (Davies and Bouldin 1976) which calculates intra and inter cluster variability and here reflects high inter-cluster and low intra-cluster variability promising a satisfactory clustering result (Levers et al. 2015). The second sensibility analysis is determined by the mean distance of the classified grid cells to the codebook vectors of the cluster they belong to, a steep increase equals an adverse compromise between reduced number of cluster and precision of cluster characteristics. A) We defined 8 clusters (4*2 hexagonal plane) as the optimum. Although DB index for 4 and 6 clusters was lower, we selected 8 clusters due the drop in mean distance which was more satisfactory.

References:

Davies, D.L. & Bouldin, D.W. (1979) A cluster separation measure. *IEEE Transaction on Pattern Analysis*

and Machine Intelligence, 1, 224–227.

Levers, C., Müller, D., Erb, K., Haberl, H., Jepsn, M.R., Metzger, P. *et al.* (2015) Archetypical patterns and trajectories of land systems in Europe. *Regional Environmental Change*, 1-18.

C. GRASSLAND TYPE BUNDLES IN GPCA EL TOKIO

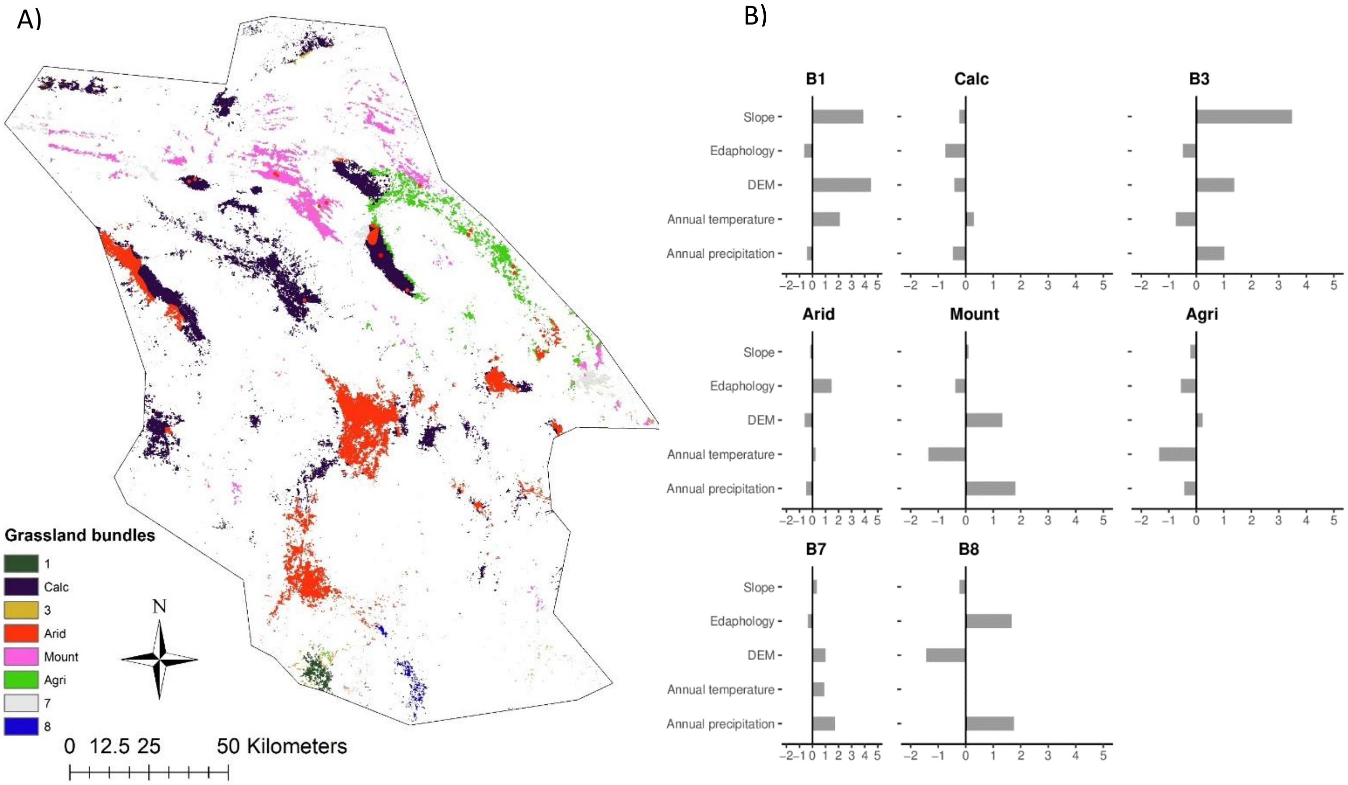

Figure 2. Fifty runs of the iterative SOM algorithm were done to discover the dominant cluster pattern and the pixel level stability in each cluster membership. (A) Once the cluster pattern is determined, the standardized code vectors were used to define what defined each cluster and define each grassland type. (B) Standardization was obtained by using z-score normalization to obtain zero mean and unit variance using decostand from vegan package. This normalization allowed us to interpret the values of

each cluster as deviations from the GPCA El Tokio averages represented by 0. This variable values are called “code vectors’ that characterize each cluster.
