# Supplementary material for: Grassland type and seasonal effects have a bigger influence on plant functional and taxonomical diversity than prairie dog disturbances in semiarid grasslands
Source: Ecol Evol. 2022 Jul 13;12(7):e9040. doi: 10.1002/ece3.9040 (PMC9279056; doi:10.1002/ece3.9040)
Supplement: Supplementary file 2 — Appendix S2 [file ECE3-12-e9040-s002.pdf]

Table S2_1. Historical and present colony data. Prairie dog colony and average burrow density for selected sites. Comparison of areas from 2002 and 2018-2019 are provided, as well as the number of colony patches and percentage lost or gained. Average burrow density per-quadrat is provided together with its standard deviation. Further an estimated number of burrows for each colony is estimated based on number of animals multiplied by the total area divided by the sampled area.

Grassland type	Colony name	Total area 2002 (ha)	Number of colony patches (2002)	Total area 2018-2019 (ha)	% Lost or gained	Number of colony patches (2018-2019)	Average burrow density per-cuadrat	SD	Number of random squares	Estimated number of burrows in whole colony	Comment
Mountain	Las Puyas	106.72	2	52.48	50.82	3.00	1.65	2.04	26.00	28933.90	
Mountain	Las Hormigas	309.7	3	267.96	13.48	6.00	1.17	1.17	29.00	104719.05	
Mountain	Tanque de Emergencia	731.89	2	554.94	24.18	6.00	0.86	1.22	30.00	462449.17	Dos Arbolitos and El Cercado adjacent colonies included
Calcareous	La Soledad	7699.54	2	6748.67	12.35	1.00	3.71	3.70	38.00	8347041.68	Alamito area of the colony is not included
Calcareous	El Uron	772.05	1	678.46	12.12	1.00	1.95	2.42	38.00	440401.26	
Calcareous	La India	2434.06	1	558.88	77.04	5.00					Google earth shows bigger colony in 2018-2019
Arid	LaTrueba	11.08	1	11.38	-2.74	1.00	2.42	1.56	12.00	9169.96	Gain most likely due to differences in delimiting the colony
Arid	El Salado 7	24.56	1	19.70	19.79	1.00	1.64	1.69	14.00	10788.10	
Arid	El Manantial	363.77	1	246.04	32.36	1.00	1.23	1.36	30.00	101150.60	
Agri	La Concha										Active at least since 2011
Agri	La Trinidad										Active at least since 2016
Agri	El Potosi										Active at least since 2011

*Colony area was obtained from historical and present delimitations of colonies (provided by the Mexican organizations PROFAUNA and Organización Visa Silvestre A.C.-OVIS).

*Number of burrows per quadrat are obtained from a thesis project by Antonia Lietschke, (2021) from TU-Dresden, Dresden, which can be requested the corresponding author. The study randomly delimited multiple 30x30 m plots in each colony and visually inspected the number of burrows based on satellite imagery from 2016-2017 based on the World Imagery Map hosted by ESRI and composed from WorldView-03 (0,.31m), WorldView-2 (0,5m) and GeoEye1 (0,46m). Burrow counts were further compared with georeferenced burrows obtained in the field. The study provides an estimate of burrow density in multiple grassland types of GPCA El Tokio.

TableS2_2. Correlation results. Results of spearman correlations between gower dissimilarities using daisy from the “cluster” *R* package version 2.1.2 and using “gawdis” from the gawdis *R* package version 0.1.3. Correlation tests were done between both matrices for each prairie dog disturbance condition (WOP and WP) in each season (wet and dry).

Correlations between gower dissimilarity matrices obtained from “cluster” and “gawdis” packages

	WOPdry	WOPdry	WPdry	WPwet
sp1	0.939721	0.946384	0.968475	0.932036
sp2	0.91777	0.943823	0.952713	0.984072
sp3	0.849129	0.963972	0.954179	0.980181
sp4	0.844774	0.933322	0.924487	0.906516
sp5	0.888502	0.934176	0.853739	0.945339
sp6	0.839199	0.930334	0.907258	0.928778
sp7	0.898084	0.968582	0.849707	0.908688
sp8	0.867596	0.934005	0.906891	0.906606
sp9	0.830314	0.911978	0.926686	0.914027
sp10	0.885366	0.931444	0.809018	0.932489
sp11	0.889895	0.941859	0.837977	0.925249
sp12	0.874739	0.936139	0.909091	0.947964
sp13	0.880488	0.932639	0.922287	0.980905
sp14	0.950174	0.928541	0.849707	0.981448
sp15	0.842857	0.936737	0.84824	0.941719
sp16	0.833798	0.908734	0.916789	0.947511
sp17	0.88885	0.937164	0.915323	0.882534
sp18	0.874042	0.94886	0.918255	0.954208
sp19	0.868641	0.930248	0.893328	0.974751
sp20	0.8777	0.942372	0.860337	0.951041
sp21	0.820732	0.9101	0.933651	0.934842
sp22	0.879443	0.945018	0.89956	0.930498
sp23	0.807666	0.957483	0.907258	0.929502
sp24	0.889895	0.937847	0.842742	0.953756
sp25	0.914286	0.934261	0.898827	0.944163
sp26	0.815157	0.936225	0.92632	0.930317
sp27	0.823868	0.932554	0.92522	0.924796
sp28	0.883449	0.938359	0.918255	0.926063
sp29	0.870557	0.967216	0.900293	0.942896
sp30	0.911498	0.940237	0.86107	0.915385
sp31	0.811847	0.931273	0.91239	0.962534
sp32	0.898432	0.924016	0.905425	0.882443
sp33	0.908014	0.926236		0.960633
sp34	0.783101	0.93981		0.976833
sp35	0.821603	0.936908		0.920362
sp36	0.939373	0.936054		0.922081
sp37	0.839721	0.973704		0.954208
sp38	0.953484	0.943482		0.981538
sp39	0.83101	0.921967		0.937466
sp40	0.889373	0.964142		0.979548
sp41	0.943206	0.942884		0.935294
sp42		0.938274		0.917466
sp43		0.892598		0.904525
sp44		0.969436		0.954932
sp45		0.93981		0.950679
sp46		0.974302		0.925792
sp47		0.936908		0.935656
sp48		0.952958		0.95819
sp49		0.96252		0.925339
sp50		0.928199		0.911674
sp51		0.94314		0.973122
sp52		0.918211		

Table S2_3. Top 3 models. Top 3 models and AICc values for all diversity variables and CWM means.

Dependent variable	Used distribution	Intercept	Grassland type	Prairie dog disturbance	Season	Grassland type: Prairie dog disturbance	Grassland type:Season	Prairie dog presence:Season	AICc
Richness	poisson	1.86	+	+	+	+	+	+	273.45
Richness	poisson	1.65	+	+	+	NA	NA	+	259.53
Richness	poisson	1.63	+	+	+	NA	NA	NA	256.80
Richness	poisson	1.57	+	NA	+	NA	NA	NA	255.87
T_Cover	Gaussian	289.69	+	+	+	+	+	+	625.05
T_Cover	Gaussian	266.03	+	+	+	NA	NA	NA	612.89
T_Cover	Gaussian	274.16	+	+	NA	NA	NA	NA	610.23
T_Cover	Gaussian	227.50	+	NA	NA	NA	NA	NA	614.55
Evenness	Beta		+	+	+	+	+	+	-9.23
Evenness	Beta		+	+	NA	NA	NA	NA	-28.63
Evenness	Beta		+	NA	+	NA	NA	NA	-27.09
Evenness	Beta		+	NA	NA	NA	NA	NA	-29.12
Fdivergence	Beta		+	+	+	+	+	+	-71.95
Fdivergence	Beta		NA	+	+	NA	NA	NA	-95.13
Fdivergence	Beta		NA	NA	+	NA	NA	NA	-96.83
Fdivergence	Beta		NA	+	NA	NA	NA	NA	-96.46
FEvenness	Beta		+	+	+	+	+	+	-18.61
FEvenness	Beta		NA	+	+	NA	NA	NA	-43.72
FEvenness	Beta		NA	NA	+	NA	NA	NA	-45.74
FEvenness	Beta		NA	+	NA	NA	NA	NA	-44.98
Frichness	Beta		+	+	+	+	+	+	-316.96
Frichness	Beta		+	+	+	+	NA	NA	-327.29
Frichness	Beta		+	+	+	NA	NA	NA	-326.52
Frichness	Beta		+	NA	+	NA	NA	NA	-328.34
Fspecialization	Beta		+	+	+	+	+	+	-106.23
Fspecialization	Beta		+	+	+	+	NA	+	-109.74
Fspecialization	Beta		+	+	+	+	NA	NA	-110.31
Fspecialization	Beta		+	+	NA	+	NA	NA	-111.29

RaoQ	lmmod-remlfalse	3.04	+	+	+	+	+	+	82.42
RaoQ	lmmod-remlfalse	3.04	+	+	+	NA	+	+	70.99
RaoQ	lmmod-remlfalse	2.95	+	+	+	NA	NA	+	63.42
RaoQ	lmmod-remlfalse	3.06	NA	+	+	NA	NA	+	56.21
CWMHeight	lmmod-remlfalse	2.74	+	+	+	+	+	+	138.48
CWMHeight	lmmod-remlfalse	2.16	NA	+	NA	NA	NA	NA	121.40
CWMHeight	lmmod-remlfalse	1.96	NA	NA	NA	NA	NA	NA	122.18
CWMHeight	lmmod-remlfalse	2.28	NA	+	+	NA	NA	+	123.74
CWMLeafarea	lmmod-remlfalse	-0.74	+	+	+	+	+	+	-53.84
CWMLeafarea	lmmod-remlfalse	-0.77	+	+	+	NA	NA	NA	-70.06
CWMLeafarea	lmmod-remlfalse	-0.90	NA	+	+	NA	NA	NA	-70.72
CWMLeafarea	lmmod-remlfalse	-0.92	NA	NA	+	NA	NA	NA	-70.14
Annual cover	lmmod-remlfalse	1.53	+	+	+	+	+	+	179.95
Annual cover	lmmod-remlfalse	1.53	+	NA	+	NA	NA	NA	158.52
Annual cover	lmmod-remlfalse	0.97	NA	+	+	NA	NA	NA	157.69
Annual cover	lmmod-remlfalse	0.90	NA	NA	+	NA	NA	NA	155.41
Perennial cover	lmmod-remlfalse	36.0503	+	+	+	+	+	+	464.23
Perennial cover	lmmod-remlfalse	24.19271	+	NA	+	NA	NA	NA	448.6364
Perennial cover	lmmod-remlfalse	26.38	+	+	NA	NA	NA	NA	447.08
Perennial cover	lmmod-remlfalse	22.33	+	NA	NA	NA	NA	NA	446.29
C3 cover	lmmod-remlfalse	2.06	+	+	+	+	+	+	130.95
C3 cover	lmmod-remlfalse	2.64	NA	+	+	NA	NA	NA	107.42
C3 cover	lmmod-remlfalse	2.72	NA	NA	+	NA	NA	NA	106.02
C3 cover	lmmod-remlfalse	2.88	NA	NA	NA	NA	NA	NA	107.46

C4 cover	lmmod-remlfalse	32.99	+	+	+	+	+	+	459.91
C4 cover	lmmod-remlfalse	25.95	+	+	+	NA	NA	NA	449.38
C4 cover	lmmod-remlfalse	25.52	+	+	NA	NA	NA	NA	446.51
C4 cover	lmmod-remlfalse	18.61	+	NA	NA	NA	NA	NA	449.40
Erect cover	lmmod-remlfalse	45.87	+	+	+	+	+	+	465.37
Erect cover	lmmod-remlfalse	37.46	+	+	+	NA	NA	NA	448.41
Erect cover	lmmod-remlfalse	38.59	+	+	NA	NA	NA	NA	445.66
Erect cover	lmmod-remlfalse	32.88	+	NA	NA	NA	NA	NA	446.37
Prostrate cover	lmmod-remlfalse	1.55	+	+	+	+	+	+	142.96
Prostrate cover	lmmod-remlfalse	1.65	+	+	NA	NA	NA	NA	125.89
Prostrate cover	lmmod-remlfalse	1.60	+	NA	NA	NA	NA	NA	123.47
Prostrate cover	lmmod-remlfalse	2.52	NA	NA	NA	NA	NA	NA	125.95
Forb cover	lmmod-remlfalse	2.12	+	+	+	+	+	+	133.63
Forb cover	lmmod-remlfalse	2.25	NA	+	+	NA	NA	NA	112.77
Forb cover	lmmod-remlfalse	2.34	NA	NA	+	NA	NA	NA	110.99
Forb cover	lmmod-remlfalse	2.14	NA	+	+	NA	NA	+	113.98
Graminoid cover	lmmod-remlfalse	32.68	+	+	+	+	+	+	456.03
Graminoid cover	lmmod-remlfalse	17.94	+	+	NA	NA	NA	NA	442.28
Graminoid cover	lmmod-remlfalse	16.83	+	NA	+	NA	NA	NA	443.43
Graminoid cover	lmmod-remlfalse	13.26	+	NA	NA	NA	NA	NA	442.22

Table S2_4. Species list per grassland type. Species list and abbreviations for all species found in GPCA El Tokio. Species presence is identified for both prairie dog conditions and across all grassland types. Species richness, unique species and proportion of unique species are provided for both conditions (WOP and WP) across all grassland types. Shared species and gamma diversity are calculated for each grassland type.

		Agricultural		Mountain		Calcareous		Arid	
**Species**	**Symbol**	**WOP**	**WP**	**WOP**	**WP**	**WOP**	**WP**	**WOP**	**WP**
Acalypha monostachya	ACMO			X	X				
Achnatherum eminens	ACEM44			X	X	X			
Acourtia nana	ACNA2			X	X				
Allionia choisyi	ALCH						X	X	X
Ambrosia confertiflora	AMCO3			X					
Aristida adscensionis	ARAD			X					
Aristida havardii	ARHA3			X	X	X	X		
Aristida pansa	ARPA9					X		X	X
Asclepias brachystephana	ASBR			X					
Asphodelus fistulosus	ASFI2			X	X				
Atriplex canescens	ATCA2	X							
Avena sativa	AVSA	X							
Bahia absinthifolia	BAAB						X	X	
Bouteloua dactyloides	BODA2			X	X	X		X	X
Bouteloua gracilis	BORG2			X	X			X	X
Bouteloua simplex	BOSI2				X				
Bouteloua uniflora	BOUN			X	X				
Bouvardia ternifolia	BOTE2			X	X				
Buddleja scoroides	BUSC	X		X	X	X			
Calylophus hartwegii sp. hartwegii	CAHAH		X	X	X	X	X	X	X
Carex potosina	CAREX				X				
Chaetopappa ericoides	CHER2			X	X				
Chamaesaracha coronopus	CHCO2					X	X	X	X
Euphorbia cinerascens	CHCI2	X	X	X	X	X	X	X	X
Euphorbia stictospora	CHST8						X	X	X
Cirsium texanum	CITE2				X				
Condalia warnockii	COWA								X
Conyza coulteri	COCO4	X			X				
Cryptantha mexicana	CRME4	X	X		X	X			
Cucurbita foetidisima	CUFO	X							
Dalea filiciformis	dafi						X		X
Dasyochloa pulchella	DAPU7			X	X	X	X	X	X
Dichondra argentea	DIAR2			X	X				
Dicranocarpus parviflorus	DIPA3	X				X	X	X	X
Dyssodia paposa	DYPA			X					
Enneapogon desvauxii	ENDE					X			
Ephedra compacta	ephco								X
Erigeron bonariensis	COBO			X	X				
Erioneuron avenaceum	ERAV			X		X			

Flaveria anomala	flan							X	X
Frankenia gypsophila	frgy		X					X	X
Gaillardia comosa	gaco							X	
Glandularia bipinnatifida	GLBIC		X	X					
Hedeoma drummondii	HEDR			X	X				
Heliopsis parvifolia	HEPA4	X		X			X		
Hoffmannseggia glauca	HOGL2			X				X	X
Hoffmannseggia watsonii	howa			X	X			X	
Houstonia wrightii	HOWR			X	X	X	X		
Hymenoxys odorata	HYOD		X	X	X				
Kallstroemia parviflora	KAPA			X		X			X
Kochia scoparia	BASC5	X							
Lepidium montanum	LEMO2	X	X						
Linum lewisii	LILE3			X	X	X			X
Loeselia coerulea	loco			X	X				
Macaeranthera tanacetifolia	MATA2	X							
Machaeranthera pinnatifida	MAPI	X	X	X	X	X	X	X	X
Menodora scabra	MESC			X	X				
Muhlenbergia arenaceae	MUAR					X			
Muhlenbergia arenicola	MUAR2			X					
Muhlenbergia phleoides	LYPH				X				
Muhlenbergia villiflora	MUVI4	X		X	X	X	X	X	X
Nama hispidum var. gypsicola	NAHI	X	X			X	X	X	
Nassella leucotricha	NALE3				X				
Nassella tenuissima	NATE3			X	X				
Nerisyrenia linnearifolia	NELI	X	X			X	X	X	X
Oenothera suffrutescens	OESU3			X		X	X	X	X
Oxalis corniculata	OXCO			X	X				
Parthenium hysterophorus	PAHY			X	X				
Physaria argyraea	LEAR3			X	X	X			
Polygala barbeyana	POBA							X	
Polygala macradenia	POMA7				X				
Portulaca mundula	POPI3			X	X			X	
Pseudognaphalium pringlei	PSPR2			X	X				
Salsola Kali	SAKA	X							
Salvia reflexa	SARE3			X	X				
Sanvitalia abertii	SAAB			X					
Sartwellia mexicana	same	X	X			X	X	X	X
Scleropogon brevifolius	SCBR2	X		X	X	X			
Selenia dissecta	SEDI2	X							
Senna bauhinioides	SEBA3					X	X	X	
Sida filicaulis	SIAB			X	X				
Solanum elaeagnifolium	SOEL	X		X	X				
Solidago velutina	SOVE6				X				
Sphaeralcea angustifolia	SPAN3			X		X		X	X
Sporobolus cryptandrus	SPCR	X							

Taraxacum officinale	TAOF			X	X				
Thymophylaa acerosa	THAC			X					
Thymophylla pentachaeta	THPE4			X	X				
Tiquilia canescens	TICA3	X	X	X	X	X	X		
Verbena canescens	VECA5			X	X			X	
Zinnia acerosa	ZIAC			X	X	X	X		
Zuloagaea bulbosa	PABU			X	X				
**Species richness**		**23**	**12**	**56**	**49**	**29**	**21**	**27**	**24**
**Unique species**		**15**	**4**	**14**	**9**	**13**	**5**	**8**	**5**
**Proportion of unique species**		**65.2**	**17.4**	**60.9**	**39.1**	**56.5**	**21.7**	**34.8**	**21.7**
**Shared species**		**8**		**41**		**16**		**19**	
**Grassland type Gamma**		**27**		**64**		**34**		**32**	

* Species symbols are based on the USDA plants database. Species that were not in the USDA plant database are shown in lower case letters.

Table S2_5. Post-hoc results. Results of linear and generalized linear mixed models to test how grassland types, prairie dog grazing and seasons relate to taxonomic, functional and CWM trait measures. Prairie dog disturbance (WP and WOP), season (wet or dry) and grassland type (Agri, Arid, Mount and Calc) were treated as fixed factors and grassland location as a random factor. The table is shown only for final models selected based on Akaike's Information Criterion (AIC). The table shows the test type Chi^2 and F-test. nDF= numerator degrees of freedom, dDF0= denominator degrees of freedom. Emmeans test p-adjust: Tukey= comparisons of levels within the variables from Tukey's HSD post-hoc test that show weak to very strong evidence of having an effect.

Explanatory variables	*nDF*	*dDF*	*Test*	Emmeans test p.adjust: Tukey	*p value*
Diversity measures					
**Richness**			**Chi2**		
Grassland type	*3*	-	*19.23*	Agri - Mount	0.00
				Arid - Mount	0.00
				Calc - Mount	0.00
Season	*1*	-	*13.619*	Dry - Wet	0.00
**Cover**			**Ftest**		
Grassland type	*3*	*8*	*21.534*	Agri - Mount	0.00
				Arid - Mount	0.00
				Calc - Mount	0.01
Prairie dog disturbance	*1*	*35*	*4.1815*	WOP - WP	*0.05*
**Evenness**			**Chi2**		
Grassland type	3	-	20.227	Agri - Mount	0.00
				Arid - Mount	0.02
**Fdiv**			**Chi2**		
Season	1	-	1.1302	Dry - Wet	ns
**Feve**			**Chi2**		
Season	1	-	1.2409	Dry - Wet	ns
**FSpe**			**Chi2**		
Grassland type	3	-	2.6884	-	R.I
Prairie dog disturbance	1	**-**	10.0014	-	R.I
Grassland type x Prairie dog disturbance	*3*	-	*31.6835*	Agri WOP - Agri WP	0.00
				Agri WOP - Arid WP	0.02
				Agri WP - Calc WP	*0.06*
**RaoQ**			**F-test**		
Prairie dog disturbance	1	34	26.501		R.I
Season	1	34	8.3085		R.I
Prairie dog disturbance x Season	*1*	*33*	*81.689*	WOP Dry - WP Dry	0.00
				WOP Dry - WOP Wet	0.00
				WOP Dry - WP Wet	0.00
				WP Dry - WOP Wet	0.04
				WP Dry - WP Wet	0.03
Traits					
**Perennial cover**					
Grassland type	*3*	*8*	*8.0027*	Agri - Mount	0.01

				Arid - Mount	0.01
**Erect cover**					
Grassland type	*3*	*8*	*13.506*	Agri - Mount	0.01
				Arid - Mount	0.00
				Calc - Mount	0.01
Prairie dog disturbance	1	35	3.5295	WOP - WP	*0.07*
**Prostrate**					
Grassland type	3	8	3.4652	Agri - Calc	*0.05744*
**Graminoid cover**					
Grassland type	*3*	*8*	*10.848*	Agri - Mount	0.01
				Arid - Mount	0.00
				Calc - Mount	0.04
Prairie dog disturbance	1	34	2.7661		R.I
Season	1	34	1.6083		R.I
Prairie dog disturbance x Season	*1*	*33*	*4.3516*	WOP Dry - WP Dry	*0.05*
**C4 cover**					
Grassland type	3	8	5.6564	Agri - Mount	0.03
				Arid - Mount	0.03
Prairie dog disturbance	1	34	5.7729	WOP - WP	0.02
**Annual cover**					
Season	*1*	*34*	*15.677*	Dry - Wet	0.00
**Forb cover**					
Season	*1*	*34*	*12.338*	Dry - Wet	0.00
**CWM Leaf area cover**					
Prairie dog disturbance	1	34	3.0352	WOP - WP	*0.0952*
Season	*1*	*34*	*6.6564*	Dry - Wet	0.01
**CWM Height**			**F-test**		
Prairie dog disturbance	1	34	3.149	WOP - WP	*0.08493*
**C3 cover**			**F-test**		
Season	1	34	-	Dry - Wet	*0.05572*

*R.I means that variable is within interaction

* ns means no significance

Table S2_6. Top 3 dominant species. Information on the top 3 dominant species per site, prairie dog disturbance condition (WOP and WP) and season (wet and dry).

SITE	DISTUR BANCE	SEASON	DOMINANT SPECIES		
**AGRI1**	WOP	Dry	*Lepidium montanum*	*Nama hispidum var. gypsicola*	*Avena sativa*
**AGRI1**	WOP	Wet	*Salsola Kali*	*Nama hispidum var. gypsicola*	*Lepidium montanum*
**AGRI1**	WP	Dry	*Tiquilia canescens*	*Scleropogon brevifolius*	*Machaeranthera pinnatifida*
**AGRI1**	WP	Wet	*Sartwellia mexicana*	*Tiquilia canescens*	*Machaeranthera pinnatifida*
**AGRI 2**	WOP	Dry	*Sporobolus cryptandrus*	*Heliopsis parvifolia*	*Muhlenbergia villiflora*
**AGRI 2**	WOP	Wet	*Sporobolus cryptandrus*	*Buddleja scoroides*	*Muhlenbergia villiflora*
**AGRI 2**	WP	Dry	*Machaeranthera pinnatifida*	*Calylophus hartwegii sp. Hartwegii*	*Sartwellia mexicana*
**AGRI 2**	WP	Wet	*Nama hispidum var. gypsicola*	*Nerisyrenia linnearifolia*	*Sartwellia mexicana*
**AGRI 3**	WOP	Dry	*Tiquilia canescens*	*Scleropogon brevifolius*	*Muhlenbergia villiflora*
**AGRI 3**	WOP	Wet	*Salsola Kali*	*Conyza coulteri*	*Tiquilia canescens*
**AGRI 3**	WP	Dry	*Hymenoxys odorata*	*Sartwellia mexicana*	*Glandularia bipinnatifida*
**AGRI 3**	WP	Wet	*Hymenoxys odorata*	*Sartwellia mexicana*	
**CALC 1**	WOP	Dry	*Zinnia acerosa*	*Aristida pansa*	*Muhlenbergia villiflora*
**CALC 1**	WOP	Wet	*Zinnia acerosa*	*Muhlenbergia villiflora*	*Aristida pansa*
**CALC 1**	WP	Dry	*Zinnia acerosa*	*Muhlenbergia villiflora*	*Calylophus hartwegii sp. Hartwegii*
**CALC 1**	WP	Wet	*Zinnia acerosa*	*Muhlenbergia villiflora*	*Houstonia wrightii*
**CALC 2**	WOP	Dry	*Achnatherum eminens*	*Physaria argyraea*	*Muhlenbergia villiflora*
**CALC 2**	WOP	Wet	*Muhlenbergia villiflora*	*Scleropogon brevifolius*	*Zinnia acerosa*
**CALC 2**	WP	Dry	*Muhlenbergia villiflora*	*Calylophus hartwegii sp. Hartwegii*	*Machaeranthera pinnatifida*
**CALC 2**	WP	Wet	*Achnatherum eminens*	*Muhlenbergia villiflora*	*Calylophus hartwegii sp. Hartwegii*
**CALC 3**	WOP	Dry	*Muhlenbergia villiflora*	*Dasyochloa pulchella*	*Sartwellia mexicana*
**CALC 3**	WOP	Wet	*Euphorbia cinerascens*	*Aristida havardii*	*Sartwellia mexicana*
**CALC 3**	WP	Dry	*Muhlenbergia villiflora*	*Aristida havardii*	*Sartwellia mexicana*
**CALC 3**	WP	Wet	*Muhlenbergia villiflora*	*Dicranocarpus parviflorus*	*Calylophus hartwegii sp. Hartwegii*
**MOUNT 1**	WOP	Dry	*Bouteloua dactyloides*	*Aristida havardii*	*Zinnia acerosa*
**MOUNT 1**	WOP	Wet	*Bouteloua dactyloides*	*Scleropogon brevifolius*	*Muhlenbergia villiflora*
**MOUNT 1**	WP	Dry	*Bouteloua dactyloides*	*Scleropogon brevifolius*	*Muhlenbergia villiflora*
**MOUNT 1**	WP	Wet	*Bouteloua dactyloides*	*Dichondra argentea*	*Muhlenbergia villiflora*
**MOUNT 2**	WOP	Dry	*Bouteloua dactyloides*	*Aristida havardii*	*Zinnia acerosa*
**MOUNT 2**	WOP	Wet	*Bouteloua dactyloides*	*Aristida havardii*	*Dasyochloa pulchella*
**MOUNT 2**	WP	Dry	*Bouteloua dactyloides*	*Muhlenbergia villiflora*	*Aristida havardii*
**MOUNT 2**	WP	Wet	*Bouteloua dactyloides*	*Aristida havardii*	*Muhlenbergia villiflora*
**MOUNT 3**	WOP	Dry	*Aristida adscensionis*	*Pseudognaphalium pringlei*	*Achnatherum eminens*
**MOUNT 3**	WOP	Wet	*Pseudognaphalium pringlei*	*Nassella leucotricha*	*Achnatherum eminens*
**MOUNT 3**	WP	Dry	*Muhlenbergia villiflora*	*Scleropogon brevifolius*	*Chaetopappa ericoides*

**MOUNT 3**	WP	Wet	*Muhlenbergia villiflora*	*Tiquilia canescens*	*Scleropogon brevifolius*
**ARID 1**	WOP	Dry	*Frankenia gypsophila*	*Dasyochloa pulchella*	*Muhlenbergia villiflora*
**ARID 1**	WOP	Wet	*Dasyochloa pulchella*	*Frankenia gypsophila*	*Flaveria anomala*
**ARID 1**	WP	Dry	*Frankenia gypsophila*	*Sphaeralcea angustifolia*	*Sartwellia mexicana*
**ARID 1**	WP	Wet	*Frankenia gypsophila*	*Euphorbia cinerascens*	*Nerisyrenia linnearifolia*
**ARID 2**	WOP	Dry	*Bouteloua gracilis*	*Sartwellia mexicana*	*Nerisyrenia linnearifolia*
**ARID 2**	WOP	Wet	*Calylophus hartwegii sp. Hartwegii*	*Nerisyrenia linnearifolia*	*Machaeranthera pinnatifida*
**ARID 2**	WP	Dry	*Calylophus hartwegii sp. Hartwegii*	*Machaeranthera pinnatifida*	*Nerisyrenia linnearifolia*
**ARID 2**	WP	Wet	*Nerisyrenia linnearifolia*	*Bouteloua gracilis*	*Hoffmannseggia glauca*
**ARID 3**	WOP	Dry	*Muhlenbergia villiflora*	*Aristida pansa*	*Dasyochloa pulchella*
**ARID 3**	WOP	Wet	*Euphorbia cinerascens*	*Calylophus hartwegii sp. Hartwegii*	*Nerisyrenia linnearifolia*
**ARID 3**	WP	Dry	*Buddleja scoroides*	*Machaeranthera pinnatifida*	*Nerisyrenia linnearifolia*
**ARID 3**	WP	Wet	*Muhlenbergia villiflora*	*Euphorbia cinerascens*	*Dicranocarpus parviflorus*

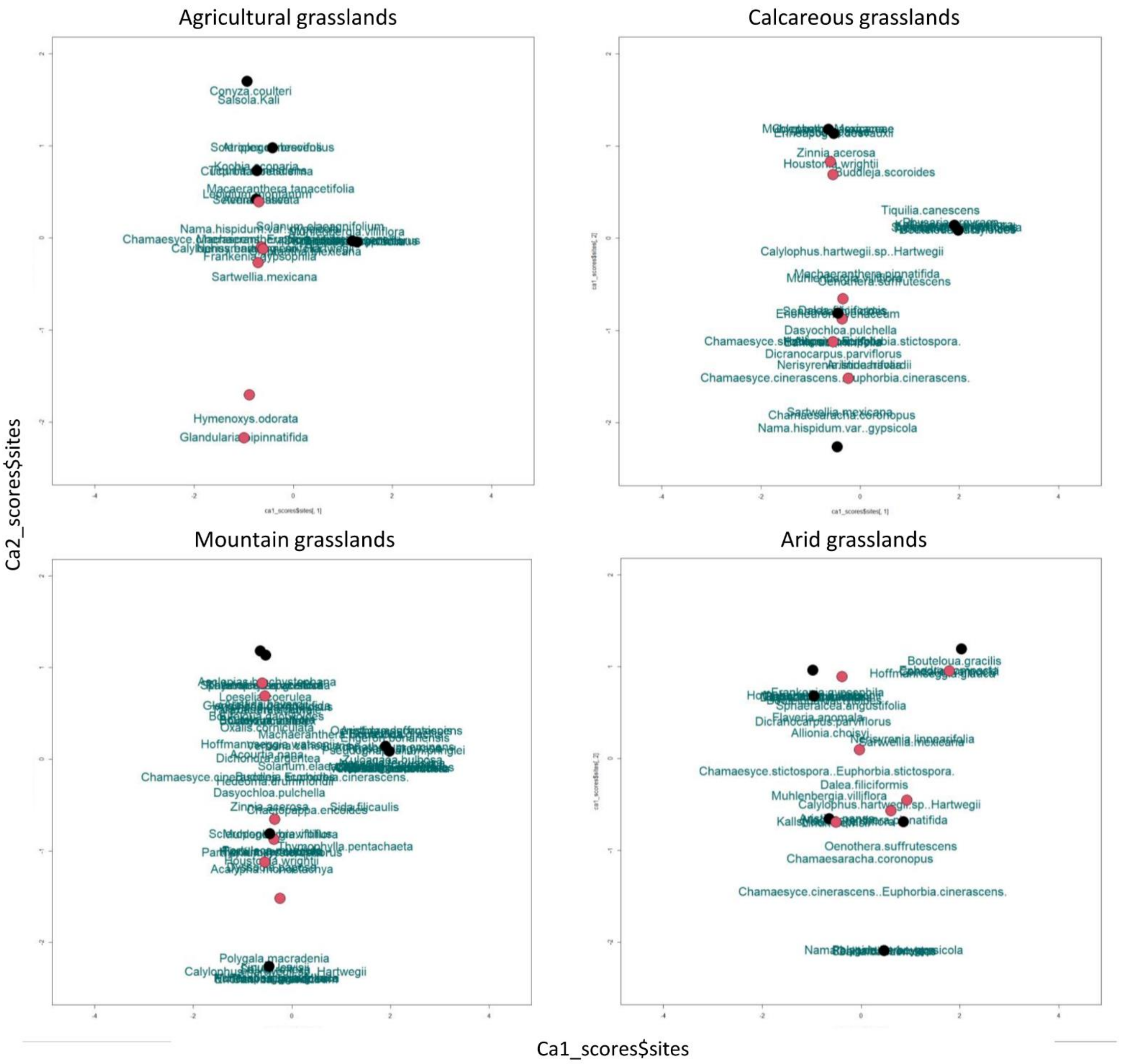

Figure S2_1. Correspondence analysis (CA) for each grassland type based on species abundance and sites. To identify dissimilarity patterns WOP and WP sites are shown in colors black and pink, respectively. The Eigenvalue/proportion explained for agricultural grassland is: CA1 = 0.93/ 21.8%, CA2 = 0.84/ 19.7%. The Eigenvalue/proportion explained for calcareous grassland is: CA1 = 0.68/ 27.8%, CA2 = 0.60/ 24.5%. The Eigenvalue/proportion explained for mountain grassland is: CA1 = 0.88/ 41.3%, CA2 = 0.38/ 17.9%. The Eigenvalue/proportion explained for arid grassland is: CA1 = 0.75/ 27.7%, CA2 = 0.57/ 21.1%.
